# Protective effects of resveratrol in animal models of chronic obstructive pulmonary disease: a preclinical systematic review and meta-analysis

**DOI:** 10.3389/fphar.2026.1854452

**Published:** 2026-08-03

**Authors:** Siping Wang, Xiaotong Wei, Yang Wang, Mengnan Zhang, Yunze Hu, Hongpeng Yu, Xize Zhang, Zheng Liang, Shaodan Hu, Li Shi

**Affiliations:** 1 Changchun University of Chinese Medicine, Changchun, China; 2 Department of Pneumology, the Affiliated Hospital to Changchun University of Chinese Medicine, Changchun, China

**Keywords:** animal models, chronic obstructive pulmonary disease, meta-analysis, preclinical studies, resveratrol

## Abstract

**Objective:**

This systematic review and meta-analysis aimed to quantitatively evaluate the protective effects of resveratrol (RES) in animal models of chronic obstructive pulmonary disease (COPD) and systematically summarize its potential molecular regulatory mechanisms.

**Methods:**

Eight databases were systematically searched for eligible animal studies from inception to February 2026. Methodological quality was assessed using the SYRCLE tool. Meta-analyses were performed using Review Manager 5.4 and Stata 18.0.

**Results:**

This meta-analysis included 14 preclinical studies involving a total of 377 experimental animals (experimental group: 239; control group: 138). The results showed that RES significantly alleviated airflow limitation and improved pulmonary function in COPD models (FEV_0.3_/FVC: SMD = 1.79, 95% CI [0.94, 2.63], *P* < 0.0001; lung compliance: SMD = 1.63, 95% CI [0.92, 2.33], *P* < 0.00001). Regarding inflammatory regulation, RES significantly reduced serum levels of pro-inflammatory cytokines, including TNF-α (SMD = −3.18, 95% CI [-4.92, −1.44], *P* = 0.0003), IL-8 (SMD = −2.79, 95% CI [-4.71, −0.86], *P* = 0.005), and IL-6 (SMD = −1.28, 95% CI [-2.03, −0.53], *P* = 0.0008). At the pulmonary level, RES also downregulated both the protein (SMD = −6.57, 95% CI [-9.94, −3.20], *P* = 0.0001) and mRNA (SMD = −1.48, 95% CI [-2.22, −0.73], *P* = 0.0001) expression of TNF-α in lung tissue. Furthermore, RES attenuated oxidative stress-related injury in lung tissue, as reflected by decreased malondialdehyde (MDA) levels (SMD = −2.64, 95% CI [-3.93, −1.35], *P* < 0.0001) and increased superoxide dismutase (SOD) activity (SMD = 3.52, 95% CI [1.22, 5.82], *P* = 0.003). Substantial heterogeneity was observed in some pooled outcomes, and Egger’s test suggested potential publication bias.

**Conclusion:**

Current preclinical evidence suggests that RES may improve pulmonary function and attenuate inflammatory responses and oxidative stress-related injury in COPD animal models. However, these findings should be considered preliminary, and further well-designed studies are needed to validate the translational relevance of RES in COPD.

**Systematic Review Registration:**

https://www.crd.york.ac.uk/PROSPERO/view/CRD420261309405, identifier CRD420261309405.

## 1 Introduction

Chronic obstructive pulmonary disease (COPD) is a heterogeneous pulmonary disorder pathologically characterized by airway remodeling and parenchymal destruction, which lead to persistent airflow limitation. It is typically triggered by exposure to noxious particles such as cigarette smoke (CS) and air pollutants (Agustí et al., 2023). COPD is currently the third leading cause of death worldwide (GBD Causes of Death Collaborators, 2025). According to the Global Burden of Disease Study, COPD caused 3.72 million deaths in 2021 (Wang et al., 2025). By 2060, the annual number of deaths from COPD and related conditions is projected to exceed 5.4 million (World Health Organization, 2022), imposing a profound burden on global public health and socioeconomic systems.

The pathogenesis of COPD is highly complex, involving inflammatory responses, oxidative stress, immune dysregulation, protease-antiprotease imbalance, and cellular senescence (Barnes et al., 2015; Wang et al., 2020; Zhou et al., 2025). At present, the clinical management of COPD primarily relies on bronchodilators and inhaled corticosteroids (ICS) (Global Initiative for Chronic Obstructive Lung Disease, 2026). Although these medications can alleviate symptoms, their ability to reverse lung function decline or prevent recurrent acute exacerbations remains limited (Roche et al., 2011). Long-term pharmacotherapy is also associated with corticosteroid resistance and adverse effects (Barnes, 2013; Lea et al., 2023). Therefore, safer and more effective therapeutic strategies are urgently needed.

Natural bioactive compounds have attracted increasing attention in COPD research because of their multi-target effects, low toxicity, and favorable safety profiles (Gonçalves and Romeiro, 2019; Li et al., 2022). Resveratrol (RES), chemically known as 3,5,4′-trihydroxy-trans-stilbene, is a natural non-flavonoid polyphenolic compound widely found in grapes, *Polygonum cuspidatum*, and peanuts (Tian and Liu, 2020). Pharmacological studies have shown that RES possesses diverse biological activities, including antioxidant (Zhu et al., 2014), anti-inflammatory (Wątroba and Szukiewicz, 2025), anti-fibrotic (Ramli et al., 2023), and anti-cancer properties (Ren et al., 2021). In respiratory disease research, RES has shown therapeutic promise (Zhu et al., 2017).

Accumulating preclinical evidence suggests that RES may attenuate COPD progression. However, existing studies vary substantially in animal strains, modeling methods, and dosing regimens, which makes direct comparison difficult. To date, no systematic quantitative synthesis has evaluated the preclinical evidence for RES in COPD animal models. Therefore, this systematic review and meta-analysis aimed to quantitatively evaluate the protective effects of RES in animal models of COPD and summarize the potential molecular mechanisms underlying its effects, thereby providing preliminary evidence for future experimental and translational research.

## 2 Materials and methods

This systematic review and meta-analysis was conducted and reported in accordance with the Preferred Reporting Items for Systematic Reviews and Meta-Analyses (PRISMA) guidelines (Page et al., 2021). The study protocol was prospectively registered in the International Prospective Register of Systematic Reviews (PROSPERO) under the registration number CRD420261309405.

### 2.1 Search strategy

Two reviewers independently conducted a comprehensive search of the following eight databases: PubMed, Embase, Web of Science, the Cochrane Library, the China National Knowledge Infrastructure (CNKI), Wanfang Database, the VIP Database (VIP), and the Chinese Biomedical Literature Database (CBM). The search covered records from the inception of each database to February 2026. The systematic search strategy combined subject headings with free-text terms. Specifically, disease-related search terms included “chronic obstructive pulmonary disease,” “COPD,” and their synonyms, while intervention-related terms included “resveratrol” and its synonyms. The detailed search strategy is provided in Supplementary Table S1. Additionally, the reference lists of the included studies and relevant reviews were manually screened to identify additional eligible studies. All retrieved records were then imported into EndNote 21 software for deduplication and preliminary screening.

### 2.2 Inclusion and exclusion criteria

The inclusion and exclusion criteria for this study were developed according to the PICOS (Population, Intervention, Comparison, Outcome, and Study design) framework.

The inclusion criteria for this meta-analysis were as follows: (1) Studies were randomized controlled animal studies (*in vivo* studies). (2) Studies used preclinical animal models of COPD, with no restrictions on species, body weight, age, sex, or modeling methods. (3) The experimental group received RES monomer (purity ≥98% or explicitly sourced from a certified standard chemical supplier), with no restrictions on the route of administration, dosage, or treatment duration (4) The control group received only a vehicle (e.g., normal saline or distilled water). or no intervention. (5) The included studies reported at least one of the following outcome measures: pulmonary function parameters [the ratio of forced expiratory volume in 0.3 s to forced vital capacity (FEV_0.3_/FVC), airway resistance (RI), dynamic compliance (Cdyn), lung compliance (CL), maximal mid-expiratory flow (MMF), and peak expiratory flow (PEF)]; inflammatory cytokines [tumor necrosis factor-α (TNF-α), interleukin-8 (IL-8), and interleukin-6 (IL-6)]; or oxidative stress indicators [malondialdehyde (MDA) and superoxide dismutase (SOD)].

The exclusion criteria were as follows: (1) Non-randomized controlled animal studies, *in vitro* studies, human clinical trials, case reports, editorials, reviews, meta-analyses, conference abstracts, duplicate publications, or studies for which full texts were unavailable. (2) Studies using non-COPD animal models or COPD models with comorbidities. (3) Studies in which the experimental group received RES derivatives or analogues, or RES in combination with other therapeutic agents. (4) Studies without a control group. (5) Studies that did not report any of the predefined outcome measures.

### 2.3 Study selection

Two reviewers independently conducted the literature screening. First, all retrieved records were imported into EndNote 21 software to remove duplicates. Subsequently, titles and abstracts were screened to exclude studies that were clearly irrelevant. Finally, the full texts of the remaining articles were retrieved and reviewed, and the final eligible studies were determined strictly according to the predefined inclusion and exclusion criteria. Any discrepancies between the two reviewers were resolved through discussion or by consulting a third reviewer.

### 2.4 Data extraction

Two reviewers independently extracted data using Microsoft Excel. The extracted data included the following information: baseline characteristics of the studies (first author’s name, publication year, and country), animal characteristics (species, body weight, age, sex, and sample size), modeling methods, treatment details (RES dose, route of administration, treatment duration, source, and purity), and outcome measures. If a study included multiple intervention groups, only data from the RES treatment group and the corresponding COPD model control group were extracted. All experimental data were recorded as the mean and standard deviation (SD). If the original study reported the standard error of the mean (SEM), it was converted to SD using a standard formula. For results presented only as graphs, data were extracted using GetData Graph Digitizer software (version 2.26). After extraction, the two reviewers cross-checked the results. Any discrepancies were resolved through discussion or by consulting a third reviewer.

### 2.5 Risk of bias assessment

Two reviewers independently assessed the risk of bias in the included studies using the SYRCLE risk of bias tool for animal studies (Hooijmans et al., 2014). The results were summarized using Review Manager 5.4 software. This tool comprises 10 specific items covering six domains of bias: selection bias, performance bias, detection bias, attrition bias, reporting bias, and other potential sources of bias. The risk of bias for each item was judged as “low risk,” “high risk,” or “unclear risk.” For studies with insufficient descriptions of randomization methods, allocation concealment, blinding procedures, or other key methodological details, we attempted to contact the corresponding authors by email for clarification. If no response was received, or if the relevant information remained unavailable, the corresponding item was judged as “unclear risk.” Any discrepancies between the two reviewers were resolved through discussion or by consulting a third reviewer.

### 2.6 Statistical analysis

All data synthesis and statistical analyses were performed using Review Manager 5.4 and Stata 18.0 software. For studies with multiple RES dose groups sharing a common control group, the sample size of the control group was divided equally among the experimental groups to avoid unit-of-analysis errors and artificial inflation of the sample size. Given that all extracted outcome measures were continuous variables, the pooled effect size was expressed as the standardized mean difference (SMD) with a 95% confidence interval (CI).

We assessed heterogeneity among the included studies using Cochran’s Q test and the *I* ^*2*^ statistic. Significant heterogeneity was defined as *I* ^*2*^ > 50% or *P* < 0.10. When significant heterogeneity was detected, a random-effects model was used; otherwise, a fixed-effects model was applied. To investigate potential sources of high heterogeneity, we conducted subgroup and sensitivity analyses. Sensitivity analysis was performed using Stata 18.0 software to evaluate the impact of individual studies on the overall pooled effect size and heterogeneity. Furthermore, potential publication bias was visually inspected using funnel plots and quantitatively assessed using Egger’s regression test in Stata 18.0. A two-tailed *P* < 0.05 was considered statistically significant for all tests, except for the Q test for heterogeneity, where *P* < 0.10 was deemed significant.

## 3 Results

### 3.1 Literature search and study selection

**FIGURE 1:**
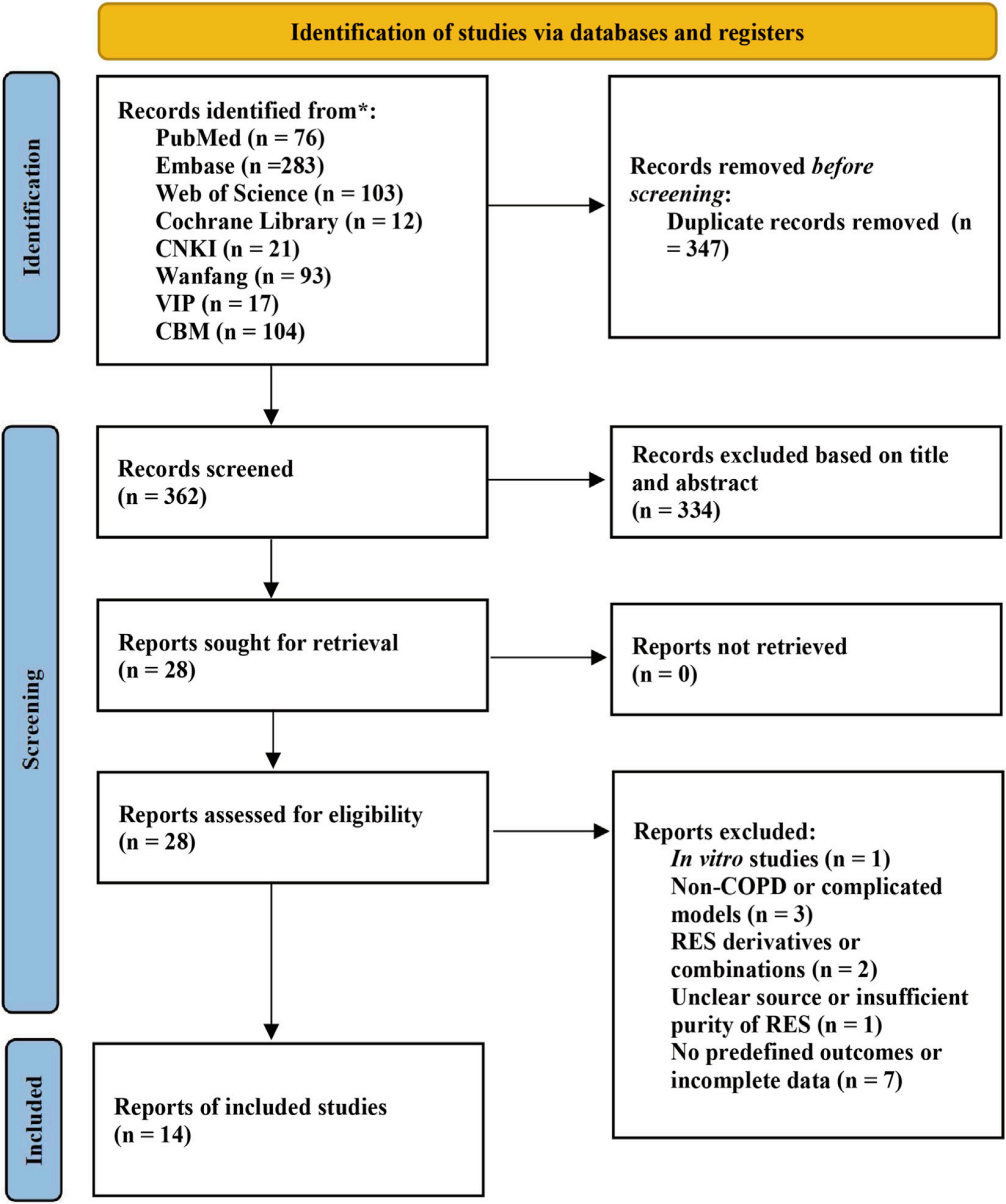
PRISMA flow diagram of the literature search and selection process.

The initial literature search identified a total of 709 records, including 76 from PubMed, 283 from Embase, 103 from Web of Science, 12 from the Cochrane Library, 21 from CNKI, 93 from Wanfang, 17 from VIP, and 104 from CBM. After removing 347 duplicate records, 362 records remained. Subsequently, titles and abstracts were screened, excluding 334 clearly irrelevant records and leaving 28 articles for full-text evaluation. Following full-text assessment, an additional 14 articles were excluded for the following reasons: *in vitro* studies (n = 1); non-COPD animal models or COPD models with comorbidities (n = 3); use of RES derivatives or analogues, or RES administered in combination with other therapeutic agents (n = 2); unclear source or insufficient purity of RES (n = 1); and lack of predefined outcome measures or incomplete outcome data (n = 7). Ultimately, a total of 14 studies met the inclusion criteria and were included in the final quantitative synthesis (Zhou et al., 2008; Şahin et al., 2011; Liu et al., 2013; Zhang et al., 2013; Li et al., 2014; Chen et al., 2016; Wang et al., 2017; Du et al., 2018; Qi et al., 2018; Zhang et al., 2020; Huo et al., 2022; Wang et al., 2023; Wu et al., 2023; Zhang et al., 2025). The literature search and study selection process is shown in Figure 1.

### 3.2 Study characteristics

This meta-analysis included 14 animal studies published between 2008 and 2025, involving 239 animals in the experimental groups and 138 animals in the control groups. All included animals were rodents. Regarding species, 11 studies used rats, including eight using Sprague-Dawley (SD) rats and three using Wistar rats. The remaining three studies used mice, including two using mice with a C57BL/6 genetic background (one using the C57BL/6J substrain) and one using Kunming mice. In terms of sex, 13 studies exclusively used male animals, whereas only one study included both males and females. The included studies employed four different modeling methods. Most studies (n = 8) established COPD models through intratracheal instillation of lipopolysaccharide (LPS) combined with continuous CS exposure. The remaining studies used CS exposure alone (n = 3), acrolein inhalation alone (n = 2), and bleomycin (BLM) administration combined with CS exposure (n = 1).

The RES dose varied widely across the included studies, ranging from 2 to 200 mg/kg. Based on the distribution of these data, RES doses were classified into three subgroups: the low-dose group (≤10 mg/kg), the medium-dose group (11–30 mg/kg), and the high-dose group (>30 mg/kg). The primary route of administration was intragastric gavage (n = 11), while a minority of studies employed aerosol inhalation (n = 1), intraperitoneal injection (n = 1), or intratracheal instillation (n = 1). Similar to dosage, the duration of RES treatment also varied substantially, ranging from 7 days to 6 months; however, the intervention period in most studies was approximately 4 weeks. The RES used in these studies was obtained from five different manufacturers: Sigma, USA (n = 9); Shanghai Kangjiu Chemical Co., Ltd., China (n = 1); Shanghai Aladdin Biochemical Technology Co., Ltd., China (n = 1); LITOZIN, USA (n = 1); and Selleck, USA (n = 1). In addition, one study used RES isolated from *Vitis amurensis* Rupr., with a reported purity of > 98%. The detailed characteristics of all included studies are summarized in Supplementary Table S2.

### 3.3 Risk of bias

**FIGURE 2:**
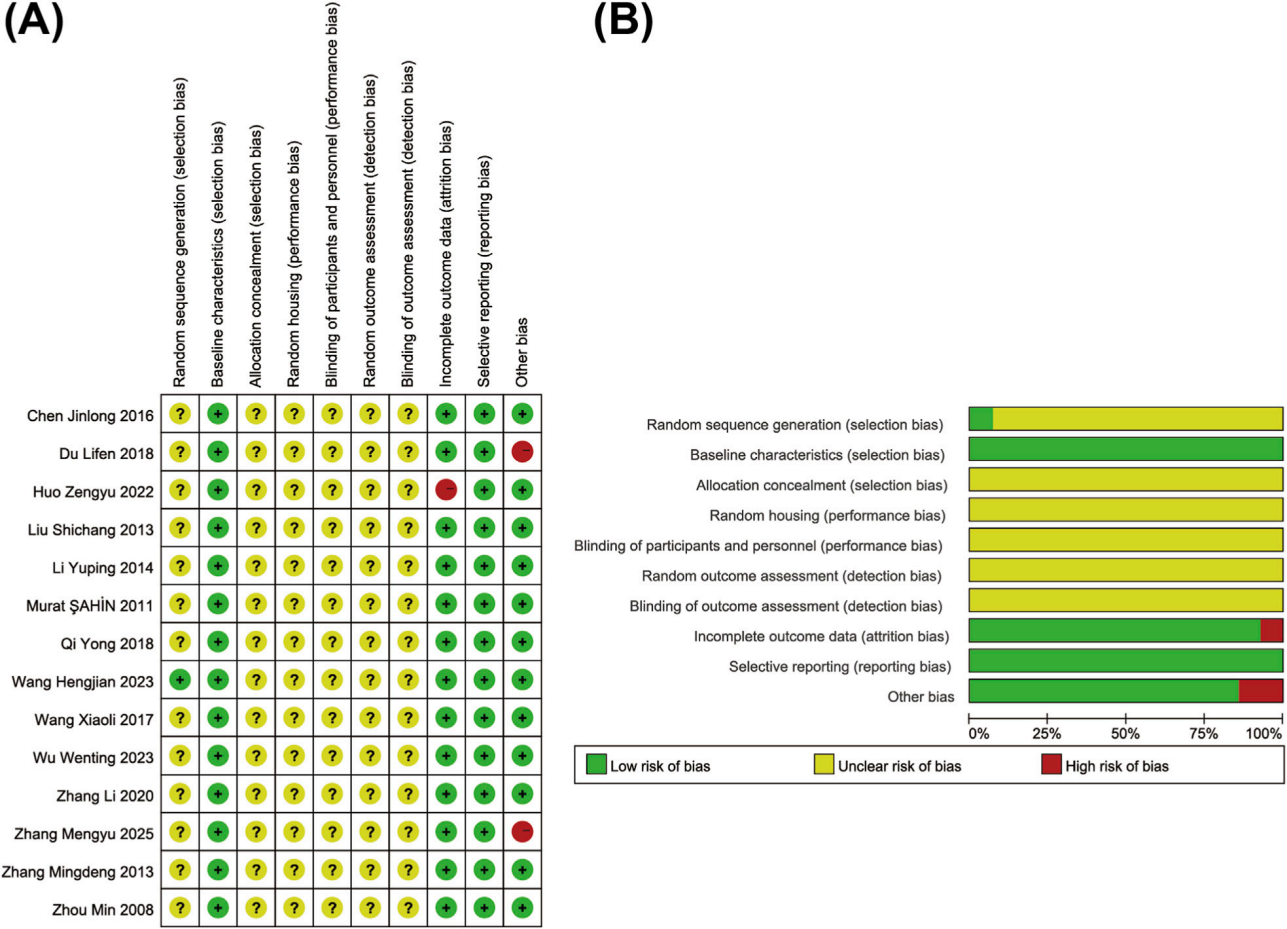
Risk of bias assessment of the included studies using the SYRCLE tool. **(A)** Risk of bias summary diagram. **(B)** Risk of bias graph diagram.

The risk of bias in all included studies was assessed using the SYRCLE risk of bias tool. The assessment results are presented in Figure 2.

For random sequence generation, 13 studies mentioned randomization but did not specify the method or sequence generation process and were therefore judged as “unclear risk.” Only one study explicitly reported using a random number table and was therefore judged as “low risk.” All included studies reported comparable baseline characteristics between the experimental and control groups and were therefore judged as “low risk.” For allocation concealment, none of the studies provided sufficient information to verify its implementation, resulting in all studies being judged as “unclear risk.” For random housing, all 14 studies maintained identical housing environments and conditions for both the experimental and control groups and were judged as “low risk.” For blinding of animal caretakers, researchers, and outcome assessors, all 14 studies failed to provide sufficient information to verify its implementation and were judged as “unclear risk.” For incomplete outcome data and selective reporting, 13 studies showed no missing data or evidence of selective reporting and were judged as “low risk.” However, one study failed to specify the reasons for animal dropouts and was therefore judged as “high risk.” For other sources of bias, two studies did not clarify whether the control group received an equivalent vehicle treatment, indicating the potential for other biases, and were judged as “high risk.” The remaining 12 studies lacked sufficient information to determine the presence of other potential biases and were judged as “unclear risk.” For items judged as “unclear risk” because of insufficient reporting, we attempted to contact the corresponding authors by email when contact information was available. However, no additional information was obtained before analysis. These findings mainly reflect incomplete reporting of key methodological details in the included animal studies. In particular, the lack of detailed information on random sequence generation, allocation concealment, and blinding limited our ability to fully assess selection, performance, and detection biases. Therefore, the pooled estimates should be interpreted with caution.

### 3.4 Meta-analysis

#### 3.4.1 Improvement of pulmonary function

Pulmonary function parameters serve as the physiological gold standard for assessing airflow limitation and disease progression in COPD. In this meta-analysis, the effects of RES on pulmonary function were assessed using the FEV_0.3_/FVC ratio and other indices of respiratory mechanics and airflow dynamics.

**FIGURE 3:**
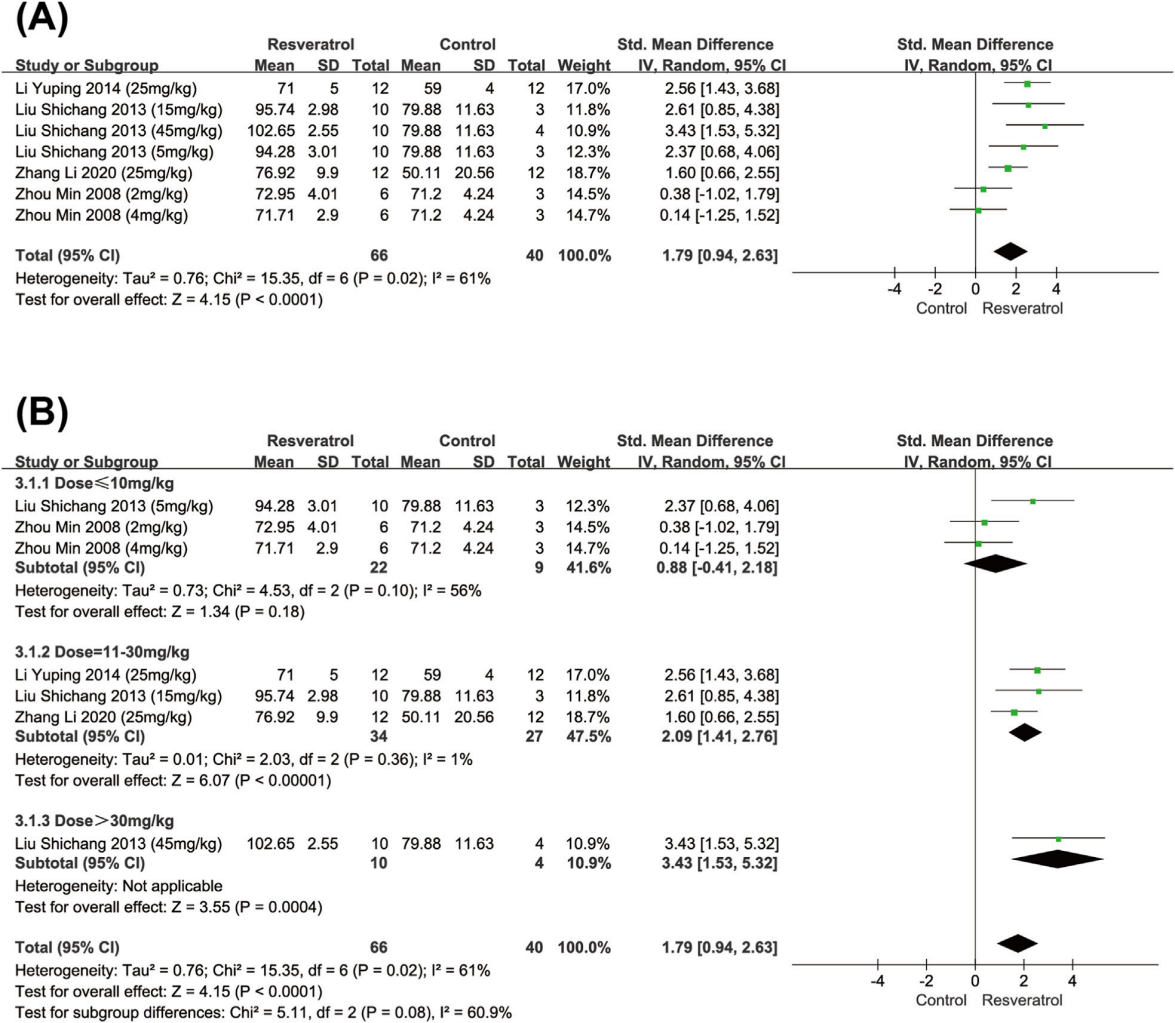
Forest plots evaluating the effect of RES on the FEV_0.3_/FVC ratio. **(A)** Overall analysis. **(B)** Subgroup analysis based on RES dose.

Four studies, comprising seven comparisons, reported the effect of RES on the FEV_0.3_/FVC ratio, and all used SD rat models. Because significant heterogeneity was observed among the included studies (*P* = 0.02, *I* ^*2*^ = 61%), a random-effects model was used for data pooling. The meta-analysis showed that RES significantly improved the FEV_0.3_/FVC ratio compared with the control group (SMD = 1.79, 95% CI [0.94, 2.63], *P* < 0.0001) (Figure 3A).

To further explore the sources of overall heterogeneity, we conducted a subgroup analysis based on the predefined RES dose subgroups (Figure 3B). The low-dose subgroup (≤10 mg/kg) showed no statistically significant improvement in pulmonary function (SMD = 0.88, 95% CI [-0.41, 2.18], *P* = 0.18, *I* ^*2*^ = 56%). In contrast, RES showed a significant improvement in the medium-dose subgroup (11–30 mg/kg) (SMD = 2.09, 95% CI [1.41, 2.76], *P* < 0.00001), and heterogeneity within the subgroup was minimal (*I* ^*2*^ = 1%). The high-dose subgroup (>30 mg/kg) showed the largest effect size (SMD = 3.43, 95% CI [1.53, 5.32], *P* = 0.0004). However, this subgroup included only one comparison, and the result should therefore be interpreted cautiously. Overall, the subgroup results suggested a potential dose-related trend as the RES dose increased. Because the test for subgroup differences did not reach conventional statistical significance (*P* = 0.08) and substantial heterogeneity remained across dose subgroups (*I* ^*2*^ = 60.9%), dosage alone may not fully explain the overall heterogeneity.

Notably, the administration routes differed among the included studies. Three studies used intragastric gavage, whereas one study used aerosol inhalation. Therefore, we further conducted a sensitivity analysis. After excluding the study that used aerosol inhalation (Zhou et al., 2008), heterogeneity was eliminated (*P* = 0.44, *I* ^*2*^ = 0%), while the pooled effect remained significant (SMD = 2.25, 95% CI [1.66, 2.84], *P* < 0.00001) (Supplementary Figure S1). This finding suggests that the administration route may have contributed to heterogeneity. Nevertheless, because of the limited number of studies, further subgroup analyses for other potential confounding factors could not be performed.

We also evaluated the effects of RES on other indices of respiratory mechanics and airflow dynamics, including RI, Cdyn, CL, MMF, and PEF. The pooled results showed that RES significantly improved CL (SMD = 1.63, 95% CI [0.92, 2.33], *P* < 0.00001), with low heterogeneity (*I* ^*2*^ = 3%) (Supplementary Figure S2C). However, no statistically significant differences were observed for RI (SMD = −2.82, 95% CI [-6.73, 1.09], *P* = 0.16), Cdyn (SMD = 4.31, 95% CI [-2.02, 10.64], *P* = 0.18), MMF (SMD = 0.26, 95% CI [-1.85, 2.37], *P* = 0.81), or PEF (SMD = 0.74, 95% CI [-0.89, 2.37], *P* = 0.37) (Supplementary Figure S2A,B,D,E). These non-significant findings should be interpreted with caution. They may be related to the limited number of included studies, small sample sizes, differences in pulmonary function testing methods or systems, and substantial between-study heterogeneity.

#### 3.4.2 Reduction of TNF-α levels

A total of nine studies evaluated the effects of RES on TNF-α levels, with samples obtained from serum, lung tissue, and bronchoalveolar lavage fluid (BALF).

**FIGURE 4:**
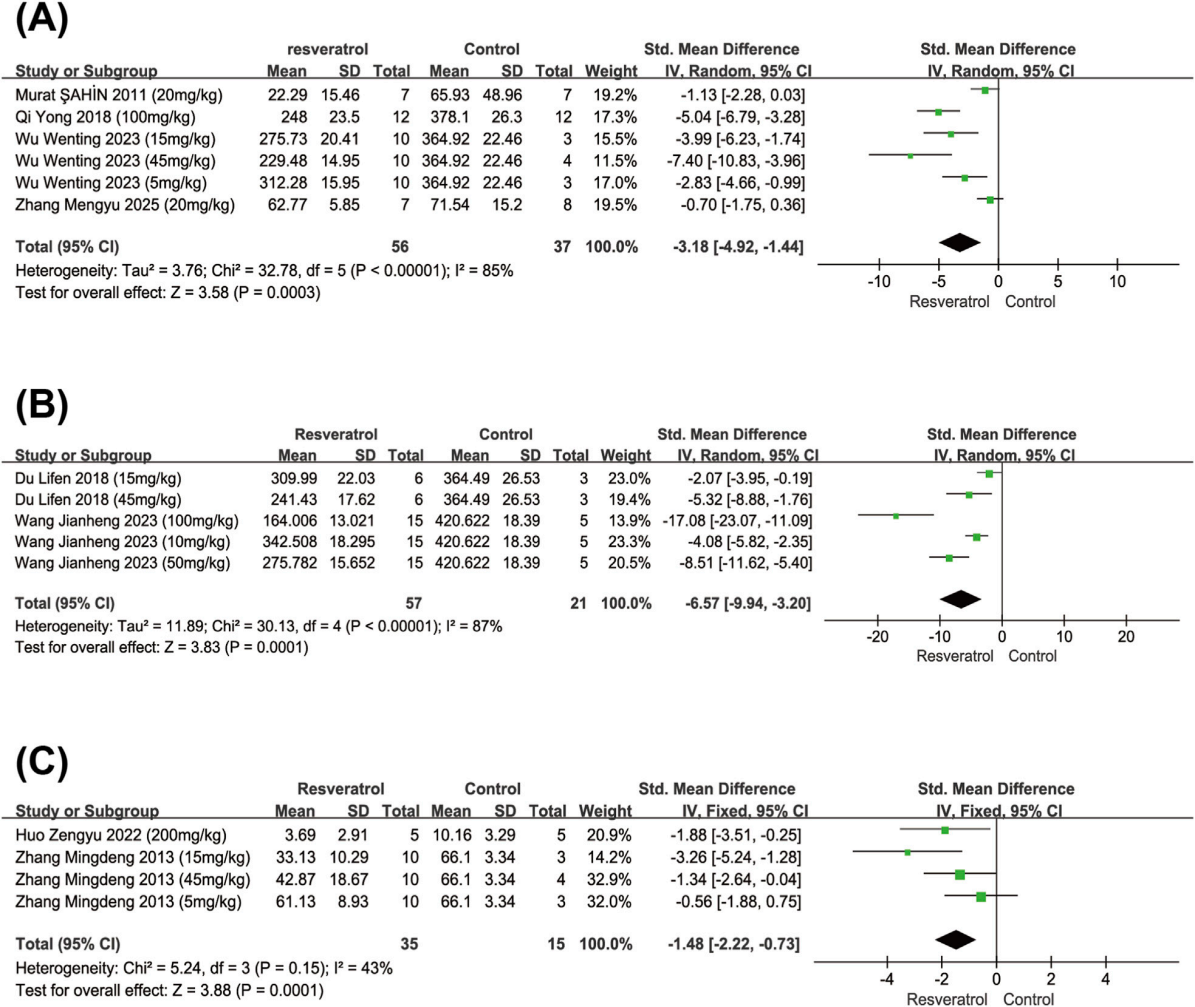
Forest plots evaluating the effects of RES on TNF-α levels and expression. **(A)** Serum TNF-α levels. **(B)** Protein expression of TNF-α in lung tissue. **(C)** Relative mRNA expression of TNF-α in lung tissue.

Pooled data from four of these studies demonstrated that, compared with the control group, RES intervention significantly reduced serum TNF-α levels (SMD = −3.18, 95% CI [-4.92, −1.44], *P* = 0.0003, *I* ^*2*^ = 85%) (Figure 4A). To further identify the potential sources of heterogeneity, we conducted a subgroup analysis based on the COPD modeling method. This analysis revealed that RES significantly decreased serum TNF-α levels in both the LPS combined with CS exposure subgroup (SMD = −4.48, 95% CI [-6.07, −2.88], *P* < 0.00001, *I* ^*2*^ = 53%) and the CS exposure alone subgroup (SMD = −0.89, 95% CI [-1.67, −0.11], *P* = 0.03, *I* ^*2*^ = 0%). The test for subgroup differences was significant (*P* < 0.0001), and within-subgroup heterogeneity was reduced (Supplementary Figure S3). This finding suggests that differences in modeling methods may partially account for the observed heterogeneity.

At the local pulmonary level, RES also reduced TNF-α expression in lung tissue at both the protein and mRNA levels. A meta-analysis of two studies showed that TNF-α protein expression in lung tissue homogenates was significantly reduced following RES treatment (SMD = −6.57, 95% CI [-9.94, −3.20], *P* = 0.0001, *I* ^*2*^ = 87%) (Figure 4B). Although this result showed high heterogeneity, the limited number of included studies precluded further subgroup analyses. Furthermore, the pooled analysis of another two studies indicated that RES significantly downregulated the relative mRNA expression of TNF-α in lung tissue (SMD = −1.48, 95% CI [-2.22, −0.73], *P* = 0.0001) (Figure 4C). Notably, the heterogeneity in the mRNA subgroup was moderate (*I* ^*2*^ = 43%); therefore, a fixed-effects model was applied. These findings suggest that RES may reduce pulmonary TNF-α expression, although the evidence remains limited by the small number of studies and the high heterogeneity observed in protein-level outcomes.

Additionally, because only one study (Chen et al., 2016) measured TNF-α levels in BALF, this outcome was not included in the quantitative analysis. This study reported that RES intervention reduced TNF-α concentrations in BALF (from 2,821 ± 988 pg/mL to 108 ± 167 pg/mL), providing qualitative support for a potential inhibitory effect of RES on local pro-inflammatory mediators within the airways. However, because this finding was derived from a single study, it should be regarded as supportive rather than confirmatory evidence.

#### 3.4.3 Reduction of IL-8 levels

A total of five studies evaluated the effects of RES on IL-8 levels, with samples obtained from serum and lung tissue.

**FIGURE 5:**
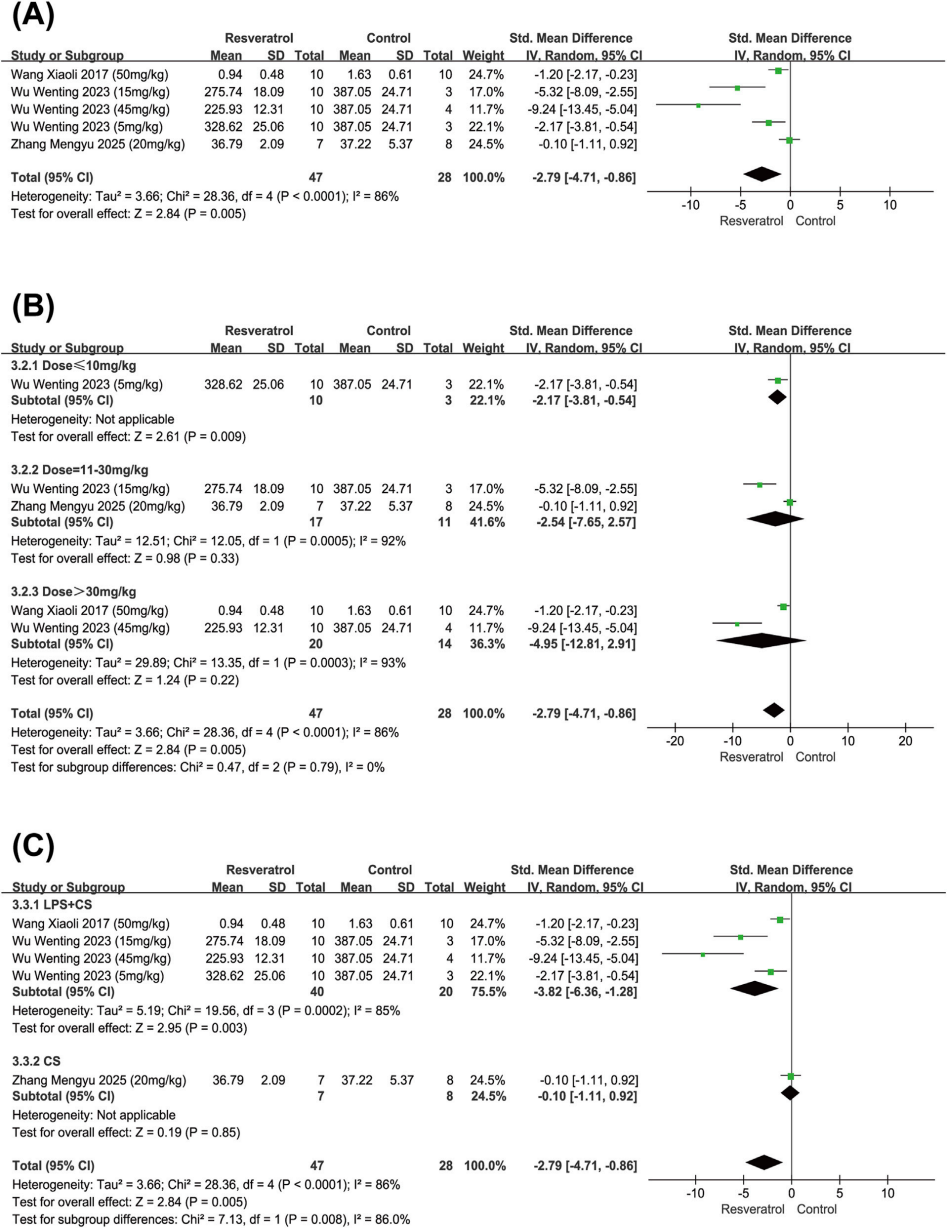
Forest plots evaluating the effects of RES on serum IL-8 levels. **(A)** Overall analysis. **(B)** Subgroup analysis based on RES dose. **(C)** Exploratory subgroup analysis based on modeling method.

Pooled data from three of these studies, comprising five comparisons, showed that, compared with the control group, RES intervention significantly reduced serum IL-8 levels (SMD = −2.79, 95% CI [-4.71, −0.86], *P* = 0.005, *I* ^*2*^ = 86%) (Figure 5A). Subgroup analysis based on RES dose did not identify the source of heterogeneity (*P* = 0.79) (Figure 5B). To further explore heterogeneity, we performed an exploratory subgroup analysis according to the COPD modeling method. The results showed a significant subgroup difference between the LPS combined with CS exposure subgroup and the CS exposure alone subgroup (*P* = 0.008) (Figure 5C). RES significantly reduced serum IL-8 levels in the LPS combined with CS exposure subgroup (SMD = −3.82, 95% CI [-6.36, −1.28], *P* = 0.003), although substantial heterogeneity remained (*I* ^*2*^ = 85%). In contrast, no significant reduction was observed in the CS exposure alone subgroup (SMD = −0.10, 95% CI [-1.11, 0.92], *P* = 0.85). However, this result should be interpreted with caution because the modeling method and animal species completely overlapped in the included studies. Specifically, all studies using LPS combined with CS exposure were conducted in rats, whereas the only study using CS exposure alone was conducted in mice. Therefore, the observed subgroup difference may reflect the combined influence of modeling method and animal species, rather than the independent effect of either factor alone. Because of the limited number of eligible studies, further subgroup analyses could not be reliably performed.

In addition to serum data, two studies evaluated the effects of RES on IL-8 levels in lung tissue. A quantitative meta-analysis was not feasible because of the limited number of studies. However, both studies reported reductions in lung tissue IL-8 levels after RES treatment. Specifically, Du et al. (2018) reported a dose-related decrease in IL-8 protein concentrations in lung homogenates (control: 276.97 ± 35.98 pg/mL; 15 mg/kg RES: 238.73 ± 36.18 pg/mL; 45 mg/kg RES: 174.37 ± 20.22 pg/mL). Furthermore, Huo et al. (2022) reported that RES significantly downregulated the mRNA expression of IL-8 in lung tissue (from 3.85 ± 0.78 to 1.59 ± 0.45).

#### 3.4.4 Reduction of IL-6 levels

A total of three studies evaluated the effects of RES on IL-6 levels, with samples obtained from serum and BALF.

**FIGURE 6:**
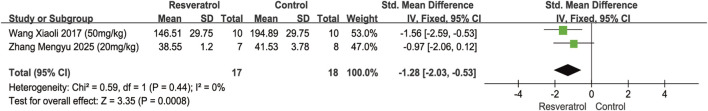
Forest plot evaluating the effects of RES on serum IL-6 levels.

Pooled data from two of these studies showed that, compared with the control group, RES intervention significantly reduced serum IL-6 levels (SMD = −1.28, 95% CI [-2.03, −0.53], *P* = 0.0008). Because no statistical heterogeneity was observed *(I* ^*2*^ = 0%), the data were pooled using a fixed-effects model (Figure 6).

In addition to serum data, one study evaluated the effect of RES on IL-6 levels in BALF. Because only a single study reported this outcome, a quantitative meta-analysis could not be performed. Chen et al. (2016) reported that RES significantly reduced IL-6 concentrations in BALF (from 2,933 ± 1991 pg/mL to 127 ± 163 pg/mL). This finding provides qualitative support for a potential inhibitory effect of RES on local IL-6 levels in the airways. However, because it was based on a single study, it should be regarded as supportive rather than confirmatory evidence.

#### 3.4.5 Regulation of oxidative stress indicators

##### 3.4.5.1 Reduction of MDA levels

**FIGURE 7:**
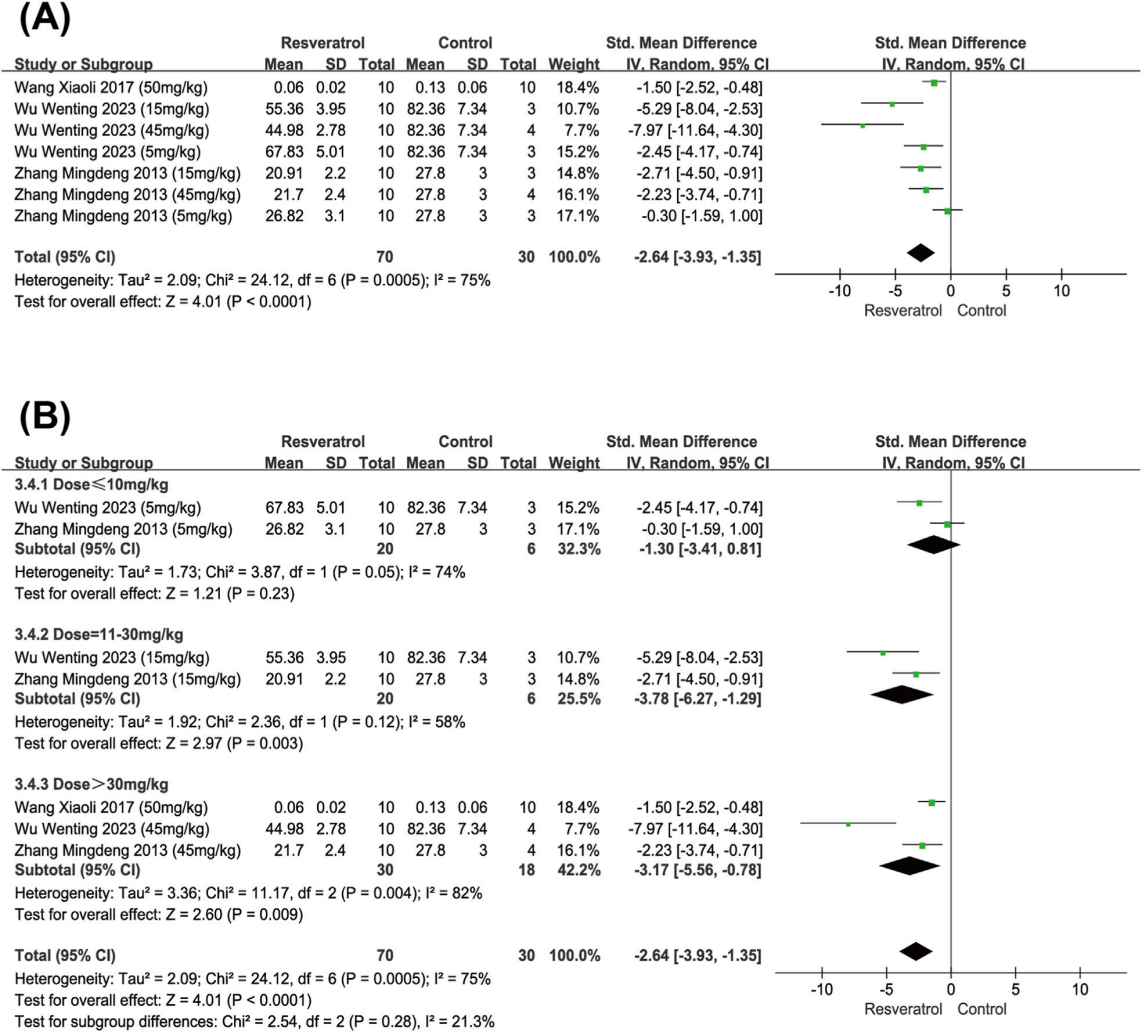
Forest plots evaluating the effects of RES on MDA levels in rat lung tissue. **(A)** Overall analysis. **(B)** Subgroup analysis based on RES dose.

A total of three studies, comprising seven comparisons, evaluated the effects of RES on MDA levels in rat lung tissue. Pooled data showed that RES intervention significantly reduced MDA levels in lung tissue (SMD = −2.64, 95% CI [-3.93, −1.35], *P* < 0.0001, *I* ^*2*^ = 75%) (Figure 7A).

Subgroup analysis based on RES dose did not fully explain the source of heterogeneity (*P* = 0.28) (Figure 7B). However, the subgroup results suggested a potential dose-related trend. Specifically, RES did not significantly reduce MDA levels in the low-dose subgroup (≤10 mg/kg) (SMD = −1.30, 95% CI [-3.41, 0.81], *P* = 0.23, *I* ^*2*^ = 74%). In contrast, RES significantly reduced MDA levels in both the medium-dose subgroup (11–30 mg/kg) (SMD = −3.78, 95% CI [-6.27, −1.29], *P* = 0.003, *I* ^*2*^ = 58%) and the high-dose subgroup (>30 mg/kg) (SMD = −3.17, 95% CI [-5.56, −0.78], *P* = 0.009, *I* ^*2*^ = 82%). Nevertheless, substantial heterogeneity persisted within these subgroups.

To further explore heterogeneity, we performed an exploratory subgroup analysis according to the COPD modeling method (Supplementary Figure S4). RES significantly reduced MDA levels in both the LPS combined with CS exposure subgroup (SMD = −3.80, 95% CI [-6.12, −1.47], *P* = 0.001, *I* ^*2*^ = 81%) and the acrolein inhalation subgroup (SMD = −1.66, 95% CI [-3.18, −0.14], *P* = 0.03, *I* ^*2*^ = 66%). However, the test for subgroup differences was not statistically significant (*P* = 0.13), suggesting that the modeling method did not fully explain the observed heterogeneity. The robustness of this subgroup result remains limited because only three original studies were included, and the acrolein inhalation subgroup was derived from a single study with multiple dose comparisons.

Additionally, one included study (Wang et al., 2017) evaluated MDA levels in BALF. Although quantitative synthesis was not feasible, this study reported that RES intervention significantly decreased MDA concentrations in the BALF of COPD rats (from 11.10 ± 2.57 nmol/mL to 5.85 ± 2.05 nmol/mL). Although this result was reported by only one study, it suggests that RES may also reduce airway oxidative stress.

##### 3.4.5.2 Restoration of SOD activity

SOD is a primary endogenous antioxidant enzyme responsible for scavenging reactive oxygen species (ROS) and serves as a crucial indicator for evaluating antioxidant defense capacity.

**FIGURE 8:**
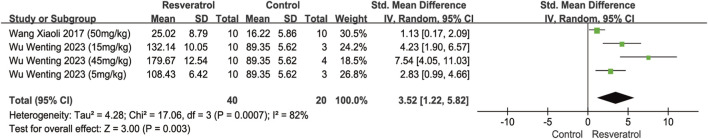
Forest plot evaluating the effects of RES on SOD activity in rat lung tissue.

A total of two studies, comprising four comparisons, evaluated the effects of RES on SOD activity in rat lung tissue. Pooled data demonstrated that, despite the presence of substantial heterogeneity (*I* ^*2*^ = 82%), RES intervention significantly increased SOD activity compared with the control group (SMD = 3.52, 95% CI [1.22, 5.82], *P* = 0.003) (Figure 8). Given the small number of included studies and the high heterogeneity, this result should be interpreted with caution.

Wang et al. (2017) also investigated SOD activity in BALF. Although these data were insufficient for quantitative synthesis, this study reported increased SOD activity in BALF after RES intervention (from 38.43 ± 5.98 U/mL to 55.75 ± 7.97 U/mL). This single-study finding suggests that RES may also increase SOD activity in BALF, but further evidence is needed.

#### 3.4.6 Sensitivity analysis

**FIGURE 9:**
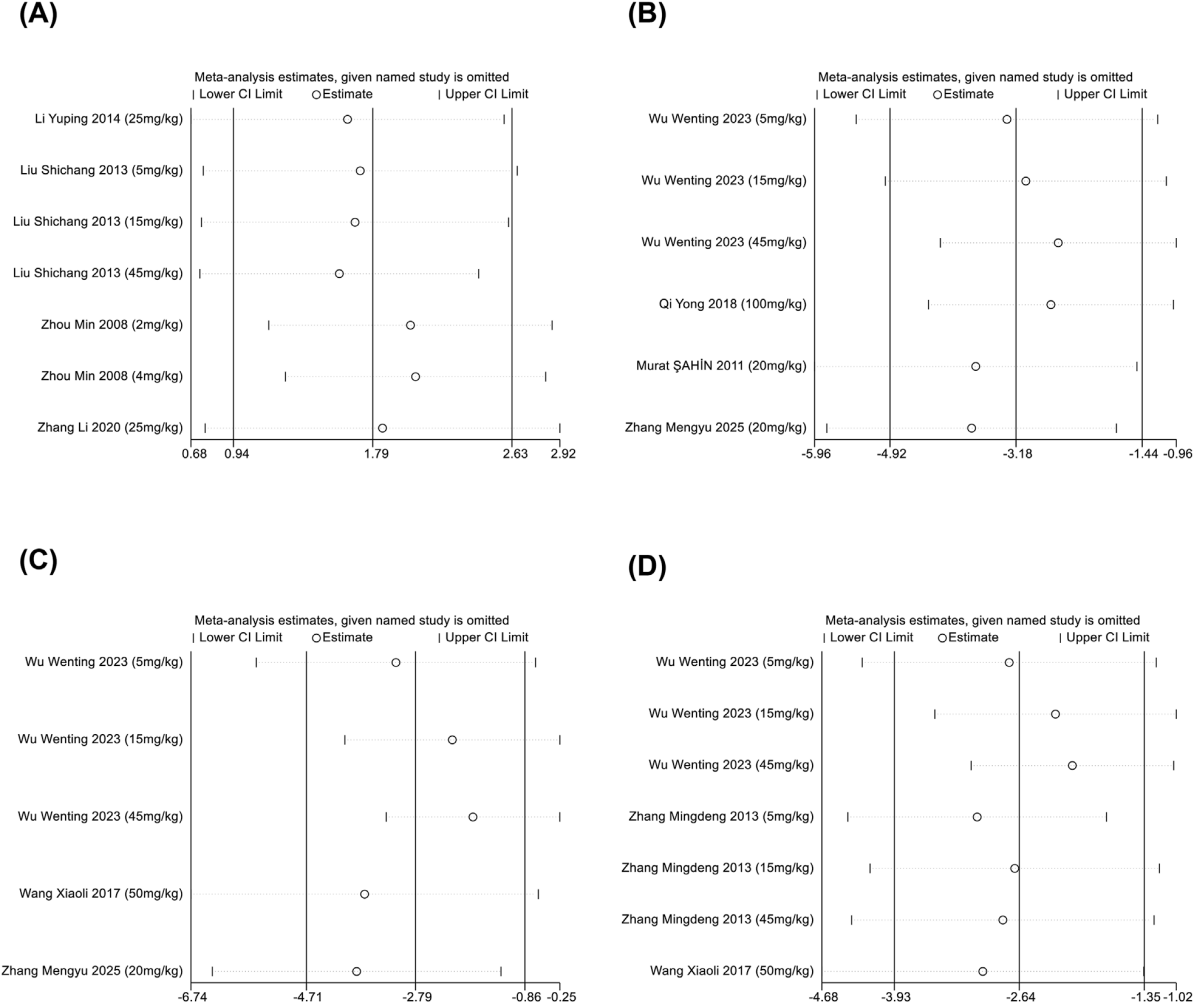
Sensitivity analysis of the primary outcomes. **(A)** FEV_0.3_/FVC. **(B)** Serum TNF-α. **(C)** Serum IL-8. **(D)** Lung tissue MDA.

To evaluate the robustness of the pooled results and explore potential sources of the observed statistical heterogeneity, we performed leave-one-out sensitivity analyses for FEV_0.3_/FVC, serum TNF-α, serum IL-8, and lung tissue MDA (Figure 9). After sequentially excluding each individual study, the direction and statistical significance of the pooled SMDs remained unchanged. In addition, all recalculated point estimates remained within the 95% CIs of the primary analyses. These findings suggest that the pooled effect estimates were generally robust in terms of effect direction and statistical significance.

#### 3.4.7 Publication bias

**FIGURE 10:**
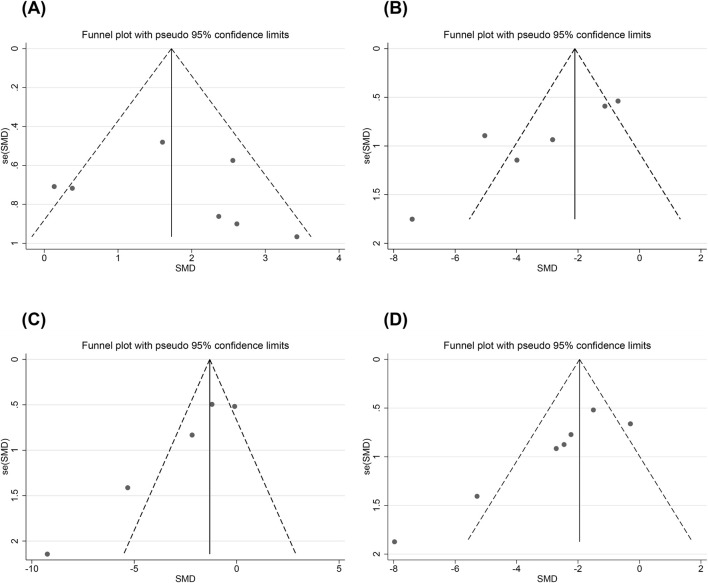
Funnel plots for evaluating potential publication bias. **(A)** FEV_0.3_/FVC. **(B)** Serum TNF-α. **(C)** Serum IL-8. **(D)** Lung tissue MDA.

To evaluate potential publication bias and small-study effects, we assessed FEV_0.3_/FVC, serum TNF-α, serum IL-8, and lung tissue MDA using funnel plots (Figure 10) and Egger’s test (Table 1). The results showed that, except for FEV_0.3_/FVC (*P* = 0.619), Egger’s test indicated significant asymmetry for the remaining outcomes (*P* < 0.05). This asymmetry may be related to small-study effects or the preferential reporting of positive results in preclinical animal studies. However, these results should be interpreted with caution because Egger’s test has limited statistical power and reliability when fewer than 10 studies are included for each outcome.

**TABLE 1:** Egger’s test.

Outcome	t	p	95% CI	
FEV_0.3_/FVC	0.53	0.619	−5.5047	8.366377
Serum TNF-α	−4.29	0.013	−9.960476	−2.138635
Serum IL-8	−5.07	0.015	−8.2621	−1.888911
Lung tissue MDA	−4.24	0.008	−7.587182	−1.86355

## 4 Discussion

### 4.1 Summary of main findings

To our knowledge, this systematic review and meta-analysis is the first comprehensive preclinical synthesis evaluating the protective effects of RES in animal models of COPD. The pooled analysis of 14 *in vivo* studies suggests that RES may exert protective effects in experimental COPD models. Physiologically, RES improved airflow limitation and respiratory mechanics, as reflected by significant increases in the FEV_0.3_/FVC ratio and CL. In terms of inflammatory regulation, RES reduced the levels of key pro-inflammatory cytokines, including TNF-α, IL-8, and IL-6, in serum, lung tissue, and BALF, suggesting a potential anti-inflammatory effect at both systemic and pulmonary levels. Regarding oxidative stress, RES may attenuate oxidative stress-related injury in COPD models, as reflected by reduced MDA levels and increased SOD activity in lung tissue.

Notably, relatively large SMD values were observed for some outcomes, particularly TNF-α protein expression in lung tissue and SOD activity. Therefore, these effect estimates should not be directly interpreted as evidence of substantial clinical efficacy. In preclinical meta-analyses, small sample sizes, small within-study standard deviations, highly controlled experimental conditions, differences in assay methods, and potential publication bias may all contribute to inflated standardized effect sizes. Taken together, the current findings support the potential protective effects of RES in COPD animal models. However, the translational value of RES requires further validation in rigorously designed preclinical studies and clinical investigations.

### 4.2 Potential molecular mechanisms

**FIGURE 11:**
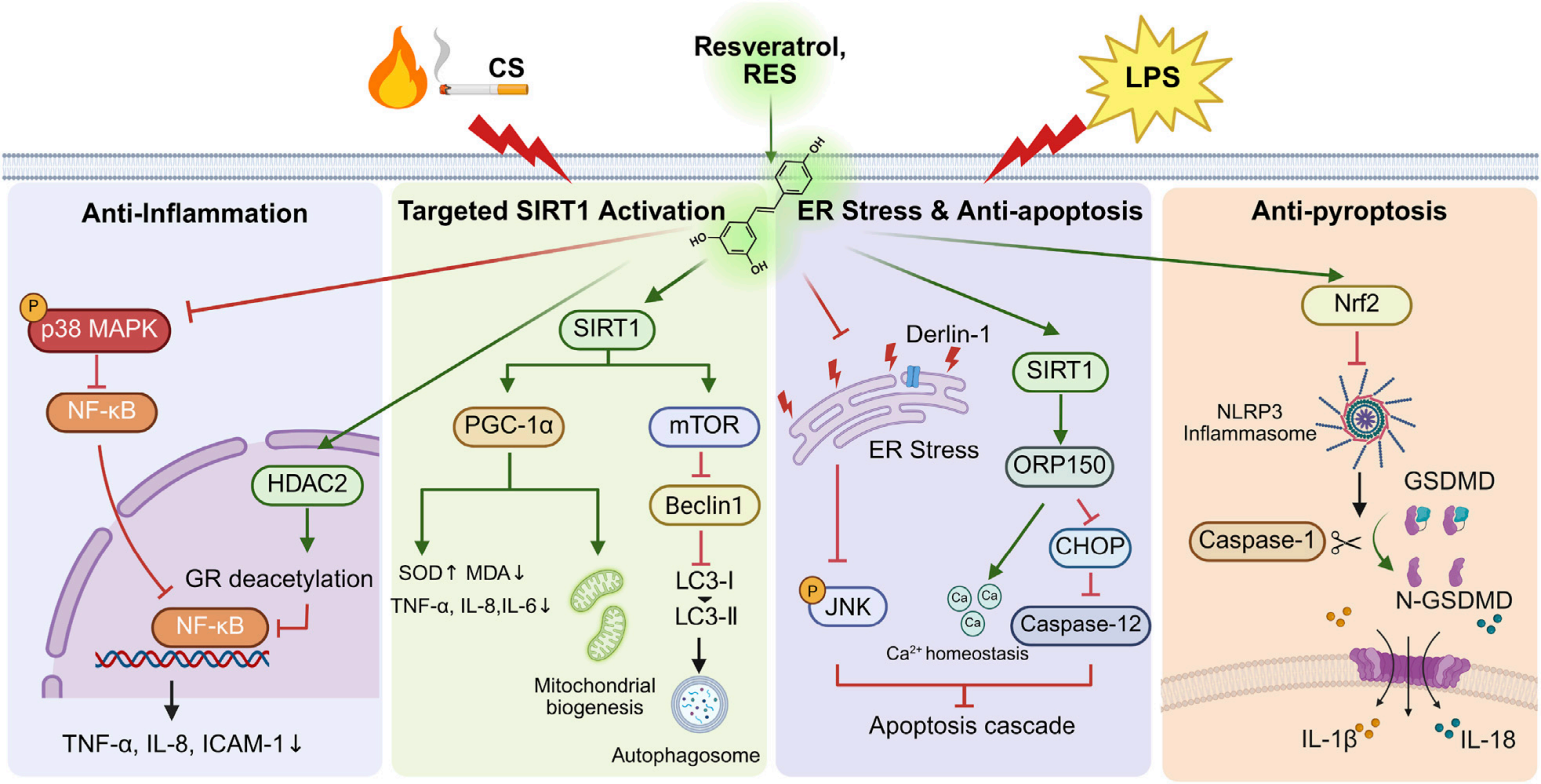
Potential molecular mechanisms underlying the protective effects of RES in COPD. RES exerts its protective effects primarily through four major mechanisms: suppressing the p38 MAPK/NF-κB inflammatory cascade and restoring the HDAC2/GR signaling axis through epigenetic regulation, thereby modulating corticosteroid resistance; targeting SIRT1 activation to modulate both PGC-1α-dependent metabolism and mTOR-mediated autophagic homeostasis; alleviating endoplasmic reticulum (ER) stress via the Derlin-1/JNK pathway and activating the SIRT1/ORP150 signaling axis to maintain cytosolic calcium homeostasis and antagonize caspase-12-dependent apoptosis; and activating the Keap1/Nrf2 antioxidant pathway to block NLRP3 inflammasome assembly and subsequent GSDMD-mediated pyroptosis. (Created with BioRender.com). Abbreviations: CHOP, C/EBP homologous protein; COPD, chronic obstructive pulmonary disease; CS, cigarette smoke; ER, endoplasmic reticulum; GR, glucocorticoid receptor; GSDMD, gasdermin D; HDAC2, histone deacetylase 2; ICAM-1, intercellular adhesion molecule-1; IL-1β, interleukin-1β; IL-6, interleukin-6; IL-8, interleukin-8; IL-18, interleukin-18; JNK, c-Jun N-terminal kinase; Keap1, Kelch-like ECH-associated protein 1; LPS, lipopolysaccharide; MDA, malondialdehyde; mTOR, mechanistic target of rapamycin; NF-κB, nuclear factor-κB; NLRP3, NOD-like receptor family pyrin domain-containing 3; Nrf2, nuclear factor erythroid 2-related factor 2; ORP150, oxygen-regulated protein 150; p38MAPK, p38 mitogen-activated protein kinase; PGC-1α, peroxisome proliferator-activated receptor-γ coactivator-1α; RES, resveratrol; SIRT1, silent information regulator 1; SOD, superoxide dismutase; TNF-α, tumor necrosis factor-α.

Based on the included studies, this review further synthesizes the potential molecular mechanisms underlying the protective effects of RES in COPD. Given that the included mechanism-related studies varied substantially in terms of modeling methods, RES administration regimens, detection techniques, and measured indicators, the data were not suitable for quantitative meta-analysis. Therefore, this section provides a qualitative synthesis of the current preclinical mechanistic evidence. The key experimental characteristics, proposed mechanisms, and corresponding measured indicators from individual studies are summarized in Table 2. An integrated schematic overview of these potential molecular mechanisms is presented in Figure 11. The translational relevance of these mechanistic targets requires further validation in rigorously designed experimental and clinical studies.

**TABLE 2:** Summary of potential mechanisms of RES in COPD animal models.

Study	Animals	Modeling method	RES administration route	Dosage	Treatment duration	Mechanism/pathway	Measured indicators and changes
Zhou et al. (2008)	Male and female SD rats (1:1)	BLM + CS exposure	Aerosol inhalation	2/4 mg/kg/d	27 days	Inflammation	ICAM-1 ↓
Şahin et al. (2011)	Male Wistar-Albino rats	CS exposure	Intraperitoneal injection	20 mg/kg/d	21 days	Inflammation	TNF-α ↓
Liu et al. (2013)	Male SD rats	Acrolein inhalation	Intragastric gavage	5/15/45 mg/kg/d	120 days	p38 MAPK/NF-κB pathway	PGE2 ↓; COX-2 ↓; NF-κB/p65 ↓; p-p38 MAPK/p38MAPK ↓
Zhang et al. (2013)	Male SD rats	Acrolein inhalation	Intragastric gavage	5/15/45 mg/kg/d	120 days	Inflammation; oxidative stress	TNF-α ↓; iNOS ↓; GSH ↑; MDA ↓
Li et al. (2014)	Male SD rats	LPS + CS exposure	Intragastric gavage	25 mg/kg/d	60 days	ER stress; apoptosis	CHOP ↓; Caspase-12 ↓
Chen et al. (2016)	Male Kunming mice	LPS + CS exposure	Intragastric gavage	50 mg/kg/d	7 days	Autophagy; inflammation	Beclin1 ↓; IL-6 ↓; IL-17 ↓; TNF-α ↓; TGF-β ↓
Wang et al. (2017)	Male Wistar rats	LPS + CS exposure	Intragastric gavage	50 mg/kg/d	20 days	SIRT1/PGC-1α pathway; inflammation; oxidative stress	IL-6 ↓; IL-8 ↓; MDA ↓; SOD ↑; SIRT1 ↑; PGC-1α ↑
Du et al. (2018)	Male Wistar rats	LPS + CS exposure	Intragastric gavage	15/45 mg/kg/d	28 days	Derlin-1/JNK pathway; ER stress; apoptosis; inflammation	IL-8 ↓; IL-13 ↓; TNF-α ↓; Derlin-1 ↓; p-JNK/JNK ↓
Qi et al. (2018)	Male SD rats	LPS + CS exposure	Intragastric gavage	100 mg/kg/d	30 days	PGC-1α-related mitochondrial biogenesis; inflammation	TNF-α ↓; PGC-1α ↑; NRF1 ↑; Tfam ↑; COX IV ↑
Zhang et al. (2020)	Male SD rats	LPS + CS exposure	Intragastric gavage	25 mg/kg/d	2 months	SIRT1/ORP150 pathway; ER stress; apoptosis	SIRT1 ↑; ORP150 ↑; CHOP ↓; Active Caspase-12 ↓; Active Caspase-3 ↓
Huo et al. (2022)	Male C57BL/6 mice	CS exposure	Intragastric gavage	200 mg/kg/d	31 days	GR/HDAC2/NF-κB pathway; inflammation	GR ↑; HDAC2 ↑; p-p65/p65 ↓; p-IκBα/IκBα ↓; IL-8 ↓; TNF-α ↓
Wang et al. (2023)	Male SD rats	LPS + CS exposure	Intragastric gavage	10/50/100 mg/kg/d	30 days	SIRT1/mTOR pathway; inflammation	IL-1β ↓; TNF-α ↓; SIRT1 ↑; mTOR ↑
Wu et al. (2023)	Male SD rats	LPS + CS exposure	Intragastric gavage	5/15/45 mg/kg/d	28 days	p38 MAPK/NF-κB pathway; inflammation; oxidative stress	TNF-α ↓; IL-8 ↓; MDA ↓; SOD ↑; MMP-9 ↓; TIMP-1 ↑; p38 MAPK ↓; IκBα ↓; NF-κB p65 ↓
Zhang et al. (2025)	Male C57BL/6J mice	CS exposure	Intratracheal instillation	20 mg/kg/d	6 months	miR-200a/Keap1/Nrf2 pathway; oxidative stress; pyroptosis; inflammation	miR-200a ↑; Keap1 ↓; Nrf2 ↑; HO-1 ↑; NQO1 ↑; GST ↑; ROS ↓; MDA ↓; SOD ↑; GSH/GSSG ↑; TXNIP ↓; NLRP3 ↓; Caspase-1 ↓; ASC ↓; GSDMD ↓; IL-1β ↓; IL-18 ↓

Abbreviations: ASC, apoptosis-associated speck-like protein containing a CARD; BLM, bleomycin; Caspase-1, cysteine aspartate-specific protease-1; Caspase-3, cysteine aspartate-specific protease-3; Caspase-12, cysteine aspartate-specific protease-12; CHOP, C/EBP, homologous protein; COPD, chronic obstructive pulmonary disease; COX-2, cyclooxygenase-2; COX IV, cytochrome c oxidase subunit IV; CS, cigarette smoke; ER, endoplasmic reticulum; GR, glucocorticoid receptor; GSDMD, gasdermin D; GSH, glutathione; GSH/GSSG, reduced glutathione/oxidized glutathione ratio; GST, glutathione S-transferase; HDAC2, histone deacetylase 2; HO-1, heme oxygenase-1; ICAM-1, intercellular adhesion molecule-1; IL-1β, interleukin-1β; IL-6, interleukin-6; IL-8, interleukin-8; IL-13, interleukin-13; IL-17, interleukin-17; IL-18, interleukin-18; iNOS, inducible nitric oxide synthase; IκBα, inhibitor of nuclear factor-κB, alpha; JNK, c-Jun N-terminal kinase; Keap1, Kelch-like ECH-associated protein 1; LPS, lipopolysaccharide; MAPK, mitogen-activated protein kinase; MDA, malondialdehyde; miR-200a, microRNA-200a; MMP-9, matrix metalloproteinase-9; mTOR, mechanistic target of rapamycin; NF-κB, nuclear factor-κB; NLRP3, NOD-like receptor family pyrin domain-containing 3; NQO1, NAD(P)H quinone oxidoreductase 1; NRF1, nuclear respiratory factor 1; Nrf2, nuclear factor erythroid 2-related factor 2; ORP150, oxygen-regulated protein 150; PGC-1α, peroxisome proliferator-activated receptor-γ coactivator-1α; PGE2, prostaglandin E2; RES, resveratrol; ROS, reactive oxygen species; SD, Sprague-Dawley; SIRT1, silent information regulator 1; SOD, superoxide dismutase; Tfam, mitochondrial transcription factor A; TGF-β, transforming growth factor-β; TIMP-1, tissue inhibitor of metalloproteinase-1; TNF-α, tumor necrosis factor-α; TXNIP, thioredoxin-interacting protein; ↑, increased or upregulated; ↓, decreased or downregulated.

#### 4.2.1 Suppression of inflammatory cascades and modulation of corticosteroid resistance

Persistent airway inflammation is a central pathological feature of COPD and contributes to disease progression. The p38 mitogen-activated protein kinase (p38 MAPK), a key signaling molecule in airway inflammation, mediates the pro-inflammatory transcription cascade from the cytoplasm to the nucleus during the pathological process of COPD (Pelaia et al., 2020). An included study reported that RES reduced p38MAPK phosphorylation and inhibited downstream nuclear factor-κB (NF-κB) activation, accompanied by downregulation of pro-inflammatory cytokines, including TNF-α and IL-8 (Wu et al., 2023). In addition, the inhibition of NF-κB transcriptional activity further downregulates the production of intercellular adhesion molecule-1 (ICAM-1), thereby suppressing the chemotaxis and excessive infiltration of neutrophils into the local lung microenvironment (Zhou et al., 2008).

In clinical practice, inhaled corticosteroid resistance is a major challenge for COPD. Its core mechanism may be related to histone deacetylase 2 (HDAC2) dysfunction, which causes defective deacetylation of the glucocorticoid receptor (GR) (Ito et al., 2006). In this context, RES shows epigenetic regulatory potential. Previous studies have reported that RES can increase the expression of GR and HDAC2 in lung tissue, promoting GR deacetylation to restore its anti-inflammatory activity (Huo et al., 2022). This process may restore the receptor’s pharmacological sensitivity to glucocorticoids, enhance their anti-inflammatory effects, and inhibit the NF-κB inflammatory pathway. These findings suggest that RES may warrant further investigation as a potential adjunctive strategy for improving corticosteroid responsiveness (Knobloch et al., 2010).

#### 4.2.2 Pleiotropic protective mechanisms mediated by targeted SIRT1 activation

As an activator of silent information regulator 1 (SIRT1) (Borra et al., 2005), RES may exert both local anti-inflammatory and systemic metabolic regulatory effects in COPD by specifically activating SIRT1 and its downstream target, peroxisome proliferator-activated receptor-γ coactivator-1α (PGC-1α). In the local airways, RES inhibits the release of inflammatory cytokines and reduces oxidative stress by activating the SIRT1/PGC-1α pathway, which helps restore pulmonary microenvironmental homeostasis (Wang et al., 2017). Furthermore, RES was reported to ameliorate mitochondrial biogenesis dysfunction and systemic inflammatory responses in rat skeletal muscle through this signaling pathway (Qi et al., 2018).

Meanwhile, SIRT1 activation plays an important role in regulating cellular autophagic homeostasis (Chun, 2015). Autophagy is an essential defense process by which cells clear pathogens and damaged organelles (Ryter et al., 2014). However, under continuous CS exposure, disrupted autophagic homeostasis can trigger excessive autophagy. This process can exacerbate inflammatory damage in lung tissue (Racanelli et al., 2018). Under physiological conditions, mechanistic target of rapamycin (mTOR) negatively regulates the Beclin1-mediated autophagic process (Kim et al., 2011; Hu et al., 2014). However, in the pathological state of COPD, this negative regulatory mechanism is disrupted. This disruption leads to abnormally high expression of the autophagy-initiating protein Beclin1 and promotes the pathological lipidation of LC3-I to LC3-II (Hwang et al., 2010; Kang et al., 2011). Based on existing mechanistic evidence, RES may activate the SIRT1/mTOR signaling axis to restore impaired mTOR activity, thereby recovering its ability to inhibit Beclin1 (Chen et al., 2016; Wang et al., 2023). This regulatory mechanism suppresses the infiltration of inflammatory cells driven by excessive autophagy and may help delay COPD progression.

#### 4.2.3 Alleviating endoplasmic reticulum stress and antagonizing apoptosis

Abnormal apoptosis of pulmonary structural cells is an important mechanism driving airway remodeling in COPD (Demedts et al., 2006). Previous studies have shown that endoplasmic reticulum (ER) stress, triggered by disrupted ER homeostasis, and its subsequent apoptotic cascades play important roles in this process (Kenche et al., 2013). When pathological stimuli, particularly CS exposure, cause excessive accumulation of unfolded or misfolded proteins that exceeds the cellular homeostatic capacity, severe ER stress can be induced, thereby initiating pro-apoptotic programs (Liu et al., 2018).

In response to this stress-induced injury, RES exhibits multi-target protective effects. In ER stress models induced by CS exposure, RES downregulated the expression of the marker protein Derlin-1 and blunted downstream c-Jun N-terminal kinase (JNK) phosphorylation, thereby inhibiting the release of TNF-α and IL-8 and alleviating alveolar epithelial cell apoptosis (Du et al., 2018). Additionally, as a SIRT1 activator, RES can upregulate the expression of oxygen-regulated protein 150 (ORP150) by activating SIRT1 (Zhang et al., 2020). Research confirms that highly expressed ORP150 antagonizes the activity of the pro-apoptotic transcription factor C/EBP homologous protein (CHOP) and downregulates the expression of the ER-specific apoptosis initiator caspase-12 (Li et al., 2014). Concurrently, ORP150 helps maintain cytosolic calcium homeostasis, which blocks calpain activation caused by calcium overload and prevents the subsequent mitochondrial apoptotic cascade (Zhang et al., 2020). Through these mechanisms, the RES-mediated SIRT1/ORP150 signaling axis relieves local protein-folding pressure in the ER, blocks lethal apoptotic crosstalk between the ER and mitochondria, and thereby preserves the structural and functional integrity of the alveolar epithelium.

#### 4.2.4 Blockade of pyroptosis

Pyroptosis, a form of programmed cell death mediated by inflammatory caspases, is involved in the airway inflammatory cascade of COPD (Feng et al., 2022). The intracellular accumulation of ROS induced by CS exposure serves as an important upstream signal that triggers the assembly and activation of the NLRP3 inflammasome. RES can activate the Keap1/Nrf2 signaling axis, promoting Nrf2 nuclear translocation and driving the transcription of downstream antioxidant genes, thereby reducing local ROS accumulation (Zhang et al., 2025).

This antioxidant effect inhibits excessive activation of the NLRP3 inflammasome, which in turn blocks the catalytic cleavage of the effector enzyme caspase-1. In the classical pyroptotic pathway, activated caspase-1 specifically cleaves the executioner protein gasdermin D (GSDMD), releasing its N-terminal fragment with membrane pore-forming activity, ultimately leading to osmotic cell lysis (Shi et al., 2015). By intervening upstream in this signaling cascade, RES reduces GSDMD cleavage and blocks the terminal execution phase of pyroptosis. Through the regulation of the Nrf2/NLRP3/GSDMD signaling pathway, RES not only maintains airway epithelial barrier integrity but also significantly inhibits the release of intracellular inflammatory mediators, such as IL-1β and IL-18, into the local pulmonary microenvironment. This process may limit the propagation of inflammatory signals and attenuate the cascade amplification of chronic airway inflammation in COPD.

### 4.3 Limitations

This systematic review and meta-analysis synthesized the evidence on the protective effects and potential mechanisms of RES in COPD animal models. However, several limitations should be considered when interpreting the current findings.

First, some key methodological details were incompletely reported in the included studies. Evaluations based on the SYRCLE tool revealed that most studies did not provide detailed reports on randomization, allocation concealment, and blinding procedures, which limited our ability to fully assess selection, performance, and detection biases. In addition, the statistical power and objectivity of the existing evidence remain limited. On the one hand, few studies meeting the inclusion criteria reported on certain secondary outcomes (e.g., IL-6 and SOD), which limited the statistical power required for detailed subgroup analyses. On the other hand, funnel plots and Egger’s test results indicate potential publication bias within this field. Given that animal experiments with non-significant or negative results are often difficult to publish, the current pooled effect sizes may overestimate the protective effects of RES to some extent. To minimize these potential biases, future preclinical studies in this field should strictly adhere to standardized experimental design and reporting guidelines.

Second, substantial heterogeneity may be partly attributable to complex experimental variables. The studies varied considerably in animal strain, modeling method, RES dose, route of administration, and intervention duration. These differences may have contributed to the heterogeneity observed in several outcomes. Although subgroup analyses were performed where feasible, residual heterogeneity remained, suggesting that the influence of these interacting factors could not be fully explained. In addition, the limited number of available studies precluded a systematic assessment of the dose-effect and time-effect relationships of RES. Moreover, some studies only reported the manufacturer of the RES reagent, without providing exact purity or batch-specific information. Therefore, differences in the source, batch, and purity of RES preparations may represent potential confounding factors and should be carefully considered when interpreting the pooled results.

Third, quantitative meta-analysis of core pathological changes, such as airway remodeling and emphysema, could not be performed. Most existing studies evaluated histopathological injury using qualitative descriptions or subjective semi-quantitative scoring methods. Due to the lack of standardized and objective morphometric data, these pathological outcomes could not be quantitatively synthesized.

Finally, laboratory-generated animal models cannot fully recapitulate the complexity of human COPD. Current rodent models are primarily used to simulate acute or subacute airway injury and do not fully replicate the complex pathophysiological processes of patients with COPD, which are characterized by a prolonged chronic course, recurrent acute exacerbations, and multisystem comorbidities. In addition, adverse events and indicators of hepatic and renal toxicity were not systematically reported in the included studies. Therefore, the safety profile of RES in COPD animal models remains insufficiently characterized. Future studies should use more clinically relevant animal models and rigorous designs to improve the reliability and translational value of preclinical evidence.

### 4.4 Conclusion

This systematic review and meta-analysis synthesized the current preclinical evidence regarding RES intervention in COPD animal models. The available evidence suggests that RES may exert protective effects by improving pulmonary function, attenuating inflammatory responses, and regulating oxidative stress-related injury. These effects may be related to multiple protective mechanisms, including suppression of inflammatory cascades, restoration of redox homeostasis, alleviation of endoplasmic reticulum stress, antagonism of apoptosis, inhibition of pyroptosis, and modulation of corticosteroid resistance via epigenetic regulation. However, due to potential methodological biases in the included studies, substantial heterogeneity in several outcomes, and insufficient safety-related evidence, the current findings should be interpreted with caution. Future high-quality preclinical studies incorporating rigorous designs, standardized outcome assessments, multiple dose levels, and varying intervention durations are needed to clarify the safety profile, optimal dosing strategy, and translational relevance of RES in COPD.

## Data Availability

The original contributions presented in the study are included in the article/Supplementary Material, further inquiries can be directed to the corresponding authors.
