# Supplementary material for: Protective effects of resveratrol in animal models of chronic obstructive pulmonary disease: a preclinical systematic review and meta-analysis
Source: Front Pharmacol. 2026 Aug 3;17:1854452. doi: 10.3389/fphar.2026.1854452 (PMC13478079; doi:10.3389/fphar.2026.1854452)
Supplement: Supplementary file 1 [file Table1.docx]

Supplementary Material

# 1 Supplementary Tables

## 1.1 Supplementary Table S1. A detailed search strategy for each database

### Search strategy in PubMed

| Search number | Query | Results |
| --- | --- | --- |
| #1 | "Pulmonary Disease, Chronic Obstructive"[Mesh] | 74545 |
| #2 | ((((((((((Pulmonary Disease, Chronic Obstructive[Title/Abstract]) OR (Chronic Obstructive Pulmonary Diseases[Title/Abstract])) OR (COPD[Title/Abstract])) OR (Chronic Obstructive Lung Disease[Title/Abstract])) OR (Chronic Obstructive Pulmonary Disease[Title/Abstract])) OR (COAD[Title/Abstract])) OR (Chronic Obstructive Airway Disease[Title/Abstract])) OR (Airflow Obstruction, Chronic[Title/Abstract])) OR (Airflow Obstructions, Chronic[Title/Abstract])) OR (Chronic Airflow Obstructions[Title/Abstract])) OR (Chronic Airflow Obstruction[Title/Abstract]) | 100246 |
| #3 | ("Pulmonary Disease, Chronic Obstructive"[Mesh]) OR (((((((((((Pulmonary Disease, Chronic Obstructive[Title/Abstract]) OR (Chronic Obstructive Pulmonary Diseases[Title/Abstract])) OR (COPD[Title/Abstract])) OR (Chronic Obstructive Lung Disease[Title/Abstract])) OR (Chronic Obstructive Pulmonary Disease[Title/Abstract])) OR (COAD[Title/Abstract])) OR (Chronic Obstructive Airway Disease[Title/Abstract])) OR (Airflow Obstruction, Chronic[Title/Abstract])) OR (Airflow Obstructions, Chronic[Title/Abstract])) OR (Chronic Airflow Obstructions[Title/Abstract])) OR (Chronic Airflow Obstruction[Title/Abstract])) | 122277 |
| #4 | "Resveratrol"[Mesh] | 12384 |
| #5 | (((((((((((((((Resveratrol[Title/Abstract]) OR (3,4',5-Stilbenetriol[Title/Abstract])) OR (3,5,4'-Trihydroxystilbene[Title/Abstract])) OR (3,4',5-Trihydroxystilbene[Title/Abstract])) OR (trans-Resveratrol[Title/Abstract])) OR (trans Resveratrol[Title/Abstract])) OR (Resveratrol-3-sulfate[Title/Abstract])) OR (Resveratrol 3 sulfate[Title/Abstract])) OR (SRT 501[Title/Abstract])) OR (SRT-501[Title/Abstract])) OR (SRT501[Title/Abstract])) OR (cis-Resveratrol[Title/Abstract])) OR (cis Resveratrol[Title/Abstract])) OR (Resveratrol, (Z)-[Title/Abstract])) OR (trans-Resveratrol-3-O-sulfate[Title/Abstract])) OR (trans Resveratrol 3 O sulfate[Title/Abstract]) | 20364 |
| #6 | ("Resveratrol"[Mesh]) OR ((((((((((((((((Resveratrol[Title/Abstract]) OR (3,4',5-Stilbenetriol[Title/Abstract])) OR (3,5,4'-Trihydroxystilbene[Title/Abstract])) OR (3,4',5-Trihydroxystilbene[Title/Abstract])) OR (trans-Resveratrol[Title/Abstract])) OR (trans Resveratrol[Title/Abstract])) OR (Resveratrol-3-sulfate[Title/Abstract])) OR (Resveratrol 3 sulfate[Title/Abstract])) OR (SRT 501[Title/Abstract])) OR (SRT-501[Title/Abstract])) OR (SRT501[Title/Abstract])) OR (cis-Resveratrol[Title/Abstract])) OR (cis Resveratrol[Title/Abstract])) OR (Resveratrol, (Z)-[Title/Abstract])) OR (trans-Resveratrol-3-O-sulfate[Title/Abstract])) OR (trans Resveratrol 3 O sulfate[Title/Abstract])) | 20752 |
| #7 | (("Pulmonary Disease, Chronic Obstructive"[Mesh]) OR (((((((((((Pulmonary Disease, Chronic Obstructive[Title/Abstract]) OR (Chronic Obstructive Pulmonary Diseases[Title/Abstract])) OR (COPD[Title/Abstract])) OR (Chronic Obstructive Lung Disease[Title/Abstract])) OR (Chronic Obstructive Pulmonary Disease[Title/Abstract])) OR (COAD[Title/Abstract])) OR (Chronic Obstructive Airway Disease[Title/Abstract])) OR (Airflow Obstruction, Chronic[Title/Abstract])) OR (Airflow Obstructions, Chronic[Title/Abstract])) OR (Chronic Airflow Obstructions[Title/Abstract])) OR (Chronic Airflow Obstruction[Title/Abstract]))) AND (("Resveratrol"[Mesh]) OR ((((((((((((((((Resveratrol[Title/Abstract]) OR (3,4',5-Stilbenetriol[Title/Abstract])) OR (3,5,4'-Trihydroxystilbene[Title/Abstract])) OR (3,4',5-Trihydroxystilbene[Title/Abstract])) OR (trans-Resveratrol[Title/Abstract])) OR (trans Resveratrol[Title/Abstract])) OR (Resveratrol-3-sulfate[Title/Abstract])) OR (Resveratrol 3 sulfate[Title/Abstract])) OR (SRT 501[Title/Abstract])) OR (SRT-501[Title/Abstract])) OR (SRT501[Title/Abstract])) OR (cis-Resveratrol[Title/Abstract])) OR (cis Resveratrol[Title/Abstract])) OR (Resveratrol, (Z)-[Title/Abstract])) OR (trans-Resveratrol-3-O-sulfate[Title/Abstract])) OR (trans Resveratrol 3 O sulfate[Title/Abstract]))) | 76 |

## Search strategy in EMBASE

| Search number | Query | Results |
| --- | --- | --- |
| #1 | 'chronic obstructive lung disease'/exp | 223686 |
| #2 | 'chronic obstructive lung disease':ab,ti OR 'chronic airflow obstruction':ab,ti OR 'chronic airway obstruction':ab,ti OR 'chronic obstructive bronchopulmonary disease':ab,ti OR 'chronic obstructive lung disorder':ab,ti OR 'chronic obstructive pulmonary disease':ab,ti OR 'chronic obstructive pulmonary disorder':ab,ti OR 'chronic obstructive respiratory disease':ab,ti OR 'chronic pulmonary obstructive disease':ab,ti OR 'chronic pulmonary obstructive disorder':ab,ti OR copd:ab,ti OR 'lung chronic obstructive disease':ab,ti OR 'lung disease, chronic obstructive':ab,ti OR 'obstructive chronic lung disease':ab,ti OR 'obstructive chronic pulmonary disease':ab,ti OR 'obstructive lung disease, chronic':ab,ti OR 'pulmonary disease, chronic obstructive':ab,ti OR 'pulmonary disorder, chronic obstructive':ab,ti | 175485 |
| #3 | #1 OR #2 | 251933 |
| #4 | 'resveratrol'/exp | 34932 |
| #5 | resveratrol:ab,ti OR '3, 4, 5 stilbenetriol':ab,ti OR '3, 4, 5 trihydroxystilbene':ab,ti OR '5 (4 hydroxystyryl) benzene 1, 3 diol':ab,ti OR 'srt 501':ab,ti OR srt501:ab,ti OR 'trans resveratrol':ab,ti OR 'trans-resveratrol':ab,ti | 24873 |
| #6 | #4 OR #5 | 37259 |
| #7 | #3 AND #6 | 283 |

## Search strategy in Web of Science

| Search number | Query | Results |
| --- | --- | --- |
| #1 | Pulmonary Disease, Chronic Obstructive (Topic) or Chronic Obstructive Pulmonary Diseases (Topic) or COPD (Topic) or Chronic Obstructive Lung Disease (Topic) or Chronic Obstructive Pulmonary Disease (Topic) or COAD (Topic) or Chronic Obstructive Airway Disease (Topic) or Airflow Obstruction, Chronic (Topic) or Airflow Obstructions, Chronic (Topic) or Chronic Airflow Obstructions (Topic) or Chronic Airflow Obstruction (Topic) | 121013 |
| #2 | Resveratrol (Topic) or 3,4',5-Stilbenetriol (Topic) or 3,5,4'-Trihydroxystilbene (Topic) or 3,4',5-Trihydroxystilbene (Topic) or trans-Resveratrol (Topic) or trans Resveratrol (Topic) or Resveratrol-3-sulfate (Topic) or Resveratrol 3 sulfate (Topic) or SRT 501 (Topic) or SRT-501 (Topic) or SRT501 (Topic) or cis-Resveratrol (Topic) or cis Resveratrol (Topic) or Resveratrol, (Z)- (Topic) or trans-Resveratrol-3-O-sulfate (Topic) or trans Resveratrol 3 O sulfate (Topic) | 32930 |
| #3 | #1 AND #2 | 103 |

## Search strategy in Cochrane Library

| Search number | Query | Results |
| --- | --- | --- |
| #1 | MeSH descriptor: [Pulmonary Disease, Chronic Obstructive] explode all trees | 8303 |
| #2 | (Pulmonary Disease, Chronic Obstructive):ti,ab,kw OR (Chronic Obstructive Pulmonary Diseases):ti,ab,kw OR (COPD):ti,ab,kw OR (Chronic Obstructive Lung Disease):ti,ab,kw OR (Chronic Obstructive Pulmonary Disease):ti,ab,kw | 26653 |
| #3 | (COAD):ti,ab,kw OR (Chronic Obstructive Airway Disease):ti,ab,kw OR (Airflow Obstruction, Chronic):ti,ab,kw OR (Airflow Obstructions, Chronic):ti,ab,kw OR (Chronic Airflow Obstructions):ti,ab,kw | 4080 |
| #4 | (Chronic Airflow Obstruction):ti,ab,kw | 1312 |
| #5 | #1 OR #2 OR #3 OR #4 | 27431 |
| #6 | MeSH descriptor: [Resveratrol] explode all trees | 431 |
| #7 | (Resveratrol):ti,ab,kw OR (3,4',5 Stilbenetriol):ti,ab,kw OR (3,5,4' Trihydroxystilbene):ti,ab,kw OR (3,4',5 Trihydroxystilbene):ti,ab,kw OR (trans Resveratrol):ti,ab,kw | 877 |
| #8 | (Resveratrol 3 sulfate):ti,ab,kw OR (SRT 501):ti,ab,kw OR (SRT501):ti,ab,kw OR (cis Resveratrol):ti,ab,kw | 15 |
| #9 | #6 OR #7 OR #8 | 877 |
| #10 | #5 AND #9 | 12 |

## Search strategy in CNKI

| Search number | Query | Results |
| --- | --- | --- |
| #1 | （主题：慢性阻塞性肺疾病 + 慢性阻塞性肺病 + 慢阻肺病 + 慢阻肺 + 慢性气道阻塞性疾病 + 慢性气流阻塞 + COPD + COAD）AND（主题：白藜芦醇 + 白黎芦醇） | 21 |

## Search strategy in VIP

| Search number | Query | Results |
| --- | --- | --- |
| #1 | ((((((((题名或关键词=慢性阻塞性肺疾病 OR 题名或关键词=慢性阻塞性肺病) OR 题名或关键词=慢阻肺病) OR 题名或关键词=慢阻肺) OR 题名或关键词=慢性气道阻塞性疾病) OR 题名或关键词=慢性气流阻塞) OR 题名或关键词=COPD) OR 题名或关键词=COAD) AND (题名或关键词=白藜芦醇 OR 题名或关键词=白黎芦醇)) | 17 |

## Search strategy in Wanfang

| Search number | Query | Results |
| --- | --- | --- |
| #1 | 主题:(慢性阻塞性肺疾病 OR 慢性阻塞性肺病 OR 慢阻肺病 OR 慢阻肺 OR 慢性气道阻塞性疾病 OR 慢性气流阻塞 OR COPD OR COAD) and 主题:(白藜芦醇 OR 白黎芦醇) | 93 |

## Search strategy in CBM

| Search number | Query | Results |
| --- | --- | --- |
| #1 | "肺疾病, 慢性阻塞性"[不加权:扩展] | 158835 |
| #2 | "慢性阻塞性肺疾病"[常用字段:智能] OR "慢性阻塞性肺病"[常用字段:智能] OR "慢阻肺病"[常用字段:智能] OR "慢阻肺"[常用字段:智能] OR "慢性气道阻塞性疾病"[常用字段:智能] OR "慢性气流阻塞"[常用字段:智能] OR "COPD"[常用字段:智能] OR "COAD"[常用字段:智能] | 214805 |
| #3 | ("慢性阻塞性肺疾病"[常用字段:智能] OR "慢性阻塞性肺病"[常用字段:智能] OR "慢阻肺病"[常用字段:智能] OR "慢阻肺"[常用字段:智能] OR "慢性气道阻塞性疾病"[常用字段:智能] OR "慢性气流阻塞"[常用字段:智能] OR "COPD"[常用字段:智能] OR "COAD"[常用字段:智能]) OR ("肺疾病, 慢性阻塞性"[不加权:扩展]) | 214805 |
| #4 | "白藜芦醇"[不加权:扩展] | 7259 |
| #5 | "白藜芦醇"[常用字段:智能] OR "白黎芦醇"[常用字段:智能] | 25337 |
| #6 | ("白藜芦醇"[常用字段:智能] OR "白黎芦醇"[常用字段:智能]) OR ("白藜芦醇"[不加权:扩展]) | 25337 |
| #7 | (("白藜芦醇"[常用字段:智能] OR "白黎芦醇"[常用字段:智能]) OR ("白藜芦醇"[不加权:扩展])) AND (("慢性阻塞性肺疾病"[常用字段:智能] OR "慢性阻塞性肺病"[常用字段:智能] OR "慢阻肺病"[常用字段:智能] OR "慢阻肺"[常用字段:智能] OR "慢性气道阻塞性疾病"[常用字段:智能] OR "慢性气流阻塞"[常用字段:智能] OR "COPD"[常用字段:智能] OR "COAD"[常用字段:智能]) OR ("肺疾病, 慢性阻塞性"[不加权:扩展])) | 104 |

## 1.2 Supplementary Table S2. Characteristics of the included studies

| **NO** | **First Author** | **Year** | **Country** | **Species** | **Weight** | **Age** | **Sex** | **Sample size (I/C)** | | **Modeling method** |
| --- | --- | --- | --- | --- | --- | --- | --- | --- | --- | --- |
| 1 | Zhou Min | 2008 | China | SD rats | 200-220 g | / | Male and female | 6/6 | 6 | Single intratracheal instillation of BLM (5 mg/kg) + CS (0.5 h/d for 27 d) |
| 2 | Murat ŞAHİN | 2011 | Turkey | Wistar-Albino rats | 250-300 g | / | Male | 7 | 7 | CS (2 h/d for 20 wk) |
| 3 | Liu Shichang | 2013 | China | SD rats | 400±25 g | / | Male | 10/10/10 | 10 | Acrolein inhalation (6.87 µg/L) for 120 d (2 h/session, twice daily) |
| 4 | Zhang Mingdeng | 2013 | China | SD rats | 400±25 g | / | Male | 10/10/10 | 10 | Acrolein inhalation (6.87 µg/L) for 120 d (2 h/session, twice daily) |
| 5 | Li Yuping | 2014 | China | SD rats | 280-320 g | 12 weeks | Male | 12 | 12 | Intratracheal instillation of LPS (2 g/L, days 1 and 31) + CS (0.5 h/d, days 2–30 and 32–60) |
| 6 | Chen Jinlong | 2016 | China | Kunming mice | / | 6-8 weeks | Male | 15 | 15 | Intratracheal instillation of LPS (20 µg, days 1 and 14) + CS (5 min/d for 30 d) |
| 7 | Wang Xiaoli | 2017 | China | Wistar rats | 250-300 g | 12-13 weeks | Male | 10 | 10 | Intratracheal instillation of LPS (200 µg, days 1 and 14) + CS (20 min/session, twice daily, days 2–13 and 15–30) |
| 8 | Du Lifen | 2018 | China | Wistar rats | 180-200 g | / | Male | 6/6 | 6 | Intratracheal instillation of LPS (200 µg, days 1 and 14) + CS (20 min/session, twice daily, days 2–13 and 15–28) |
| 9 | Qi Yong | 2018 | China | SD rats | 200±20 g | / | Male | 12 | 12 | Intratracheal instillation of LPS + CS (29 d) |
| 10 | Zhang Li | 2020 | China | SD rats | 250-300 g | 12-13 weeks | Male | 12 | 12 | Intratracheal instillation of LPS (200 µg, days 1 and 31) + CS (30 min/d, days 2–30 and 32–60) |
| 11 | Huo Zengyu | 2022 | China | C57BL/6 mice | ~ 13 g | 3-4 weeks | Male | 5 | 5 | CS (30 min/session, 4 times/d, 5 d/wk for 6 months) |
| 12 | Wang Jianheng | 2023 | China | SD rats | 300 g | 3 months | Male | 15/15/15 | 15 | Intratracheal instillation of LPS + CS (30 d) |
| 13 | Wu Wenting | 2023 | China | SD rats | 200±20 g | / | Male | 10/10/10 | 10 | Intratracheal instillation of LPS (40 µg, days 1 and 14) + CS (1 h/d for 28 d) |
| 14 | Zhang Mengyu | 2025 | China | C57BL/6J mice | 18-20 g | 7 weeks | Male | 7 | 8 | CS (5 d/wk for 6 months) |

Continued Supplementary Table S2. Characteristics of the included studies

| **NO** | **First Author** | **Treatment (I/C)** | | **RES Administration route** | **RES sources** | **Purity of RES** | **Treatment duration** | **Specimen** | **Outcome** |
| --- | --- | --- | --- | --- | --- | --- | --- | --- | --- |
| 1 | Zhou Min | RES 2/4 mg/kg/d | Vehicle | Aerosol inhalation | Shanghai Kangjiu Chemical Co., Ltd., China | > 98% | 27 days | / | ①④ ⑤ ⑥ |
| 2 | Murat ŞAHİN | RES 20 mg/kg/d | DMSO | Intraperitoneal injection | Sigma, USA | purity not specified | 21 days | Serum | ⑦ |
| 3 | Liu Shichang | RES 5/15/45 mg/kg/d | Distilled water | Intragastric gavage | Sigma, USA | purity not specified | 120 days | / | ①④ ⑤ ⑥ |
| 4 | Zhang Mingdeng | RES 5/15/45 mg/kg/d | Distilled water | Intragastric gavage | Sigma, USA | purity not specified | 120 days | Lung tissue | ⑦ ⑩ |
| 5 | Li Yuping | RES 25 mg/kg/d | 0.5% CMC-Na | Intragastric gavage | Sigma, USA | purity not specified | 60 days | / | ① ②③ |
| 6 | Chen Jinlong | RES 50 mg/kg/d | Vehicle | Intragastric gavage | Isolated from Vitis amurensis Rupr. | > 98% | 7 days | BALF | ⑦ ⑨ |
| 7 | Wang Xiaoli | RES 50 mg/kg/d | 0.5% CMC-Na | Intragastric gavage | Sigma, USA | purity not specified | 20 days | Serum; Lung tissue; BALF | ⑧⑨ ⑩ ⑪ |
| 8 | Du Lifen | RES 15/45 mg/kg/d | / | Intragastric gavage | Shanghai Aladdin Biochemical Technology Co., Ltd., China | > 99% | 28 days | Lung tissue | ⑦⑧ |
| 9 | Qi Yong | RES 100 mg/kg/d | NS | Intragastric gavage | LITOZIN, USA | purity not specified | 30 days | Serum | ⑦ |
| 10 | Zhang Li | RES 25 mg/kg/d | 0.5% CMC-Na | Intragastric gavage | Sigma, USA | purity not specified | 2 months | / | ①② ③ |
| 11 | Huo Zengyu | RES 200 mg/kg/d | DMSO | Intragastric gavage | Sigma, USA | purity not specified | 31 days | Lung tissue | ⑦⑧ |
| 12 | Wang Jianheng | RES 10/50/100 mg/kg/d | NS | Intragastric gavage | Sigma, USA | purity not specified | 30 days | Lung tissue | ⑦ |
| 13 | Wu Wenting | RES 5/15/45 mg/kg/d | NS | Intragastric gavage | Sigma, USA | purity not specified | 28 days | Serum; Lung tissue | ⑦⑧ ⑩ ⑪ |
| 14 | Zhang Mengyu | RES 20 mg/kg/d | / | Intratracheal instillation | Selleck, USA | purity not specified | 6 months | Serum | ⑦⑧⑨ |

Abbreviations: RES, resveratrol; I/C, intervention/control group; SD, Sprague-Dawley; BLM, bleomycin; CS, cigarette smoke; DMSO, dimethyl sulfoxide; LPS, lipopolysaccharide; CMC-Na, sodium carboxymethyl cellulose; NS, normal saline; BALF, bronchoalveolar lavage fluid.

Outcome: ① The ratio of forced expiratory volume in 0.3 seconds to forced vital capacity (FEV_0.3_/FVC); ② Airway resistance (RI); ③ Dynamic compliance (Cdyn); ④ Lung compliance (CL); ⑤ Maximal mid-expiratory flow (MMF); ⑥ Peak expiratory flow (PEF); ⑦ Tumor necrosis factor-α (TNF-α); ⑧ Interleukin-8 (IL-8); ⑨ Interleukin-6 (IL-6); ⑩ Malondialdehyde (MDA); ⑪ Superoxide dismutase (SOD).

# 2 Supplementary Figures

## 2.1 Supplementary Figure S1

**Supplementary Figure S1.** Forest plot evaluating the effect of RES on the FEV_0.3_/FVC ratio after excluding the study using aerosol inhalation.

## 2.2 Supplementary Figure S2

**Supplementary Figure S2.** Forest plots evaluating the effects of RES on other indices of respiratory mechanics and airflow dynamics. (A) RI. (B) Cdyn. (C) CL. (D) MMF. (E) PEF.

## 2.3 Supplementary Figure S3

**Supplementary Figure S3.** Subgroup analysis of the effects of RES on serum TNF-α levels based on modeling method.

## 2.4 Supplementary Figure S4

**Supplementary Figure S4.** Exploratory subgroup analysis of the effects of RES on MDA levels in rat lung tissue based on modeling method.
